# Supplementary material for: Harnessing clonal gametes in hybrid crops to engineer polyploid genomes
Source: Nat Genet. 2024 May 13;56(6):1075–9. doi: 10.1038/s41588-024-01750-6 (PMC11176054; doi:10.1038/s41588-024-01750-6)
Supplement: Supplementary file 1 — Supplementary Notes 1 and 2, Figs. 1−36 and Tables 1−9. [file 41588_2024_1750_MOESM1_ESM.pdf]

nature genetics

Brief Communication https://doi.org/10.1038/s41588-024-01750-6

Harnessing clonal gametes in hybrid crops to engineer polyploid genomes

In the format provided by the
authors and unedited

Contents

Supplementary Note 1-2

Supplementary Figures 1-36

Supplementary Tables 1-9

Supplementary Note 1. ***Mitosis instead of Meiosis*** **(*MiMe*) system in inbred tomato.**

The *Mitosis instead of Meiosis (MiMe)* system previously established in Arabidopsis and rice leads to clonal, unreduced gametes[1–3]. In Arabidopsis, *MiMe* was established through the mutation of three meiotic genes involved in meiotic recombination initiation (*AtSPO11-1*), sister chromatid segregation (*AtREC8*) and cell cycle control (*AtOSD1*)[2,3], whereas in rice *OsPAIR1* was targeted to ablate recombination[1]. Both Arabidopsis and rice have two *OSD1* genes, and genetic analysis in Arabidopsis has shown that double mutants (*osd1 uvi4*) undergo embryonic lethality[4]. However, many plant species, including tomato, have only a single, likely-essential, *OSD1* gene copy[1,5]. Alternative mutants that skip the second meiotic division in Arabidopsis, including *tam* (loss of function of *CYCA1;2*)[6] and a *tdm1*[7] point mutant, have yet to be explored in crops.

We set out to identify genetic factors that control entry to the second meiotic division in tomato. We confirmed that OSD1/UVI4 (the plant-specific inhibitors of the anaphase-promoting complex/cyclosome (APC/C)) are single-copy genes in tomato (Solyc10g080400), unlike Arabidopsis and rice where the function is split between two genes (Supplementary Fig.1). Meanwhile, there are nine A-type cyclins in tomato with only a single cyclin A1 gene (*SlTAM*, Solyc11g005090), with high similarity to Arabidopsis *AtCYCA1;2*[7,8] and a predicted specific role in meiosis (Supplementary Fig.2). Two different CRISPR/Cas9 constructs targeting *SlOSD1* (Supplementary Fig.3 and Supplementary Table 1) and one CRISPR/Cas9 construct targeting *SlTAM* (Extended Data Fig.1a and Supplementary Table 1, 2) were generated and transformed into the tomato cultivar Micro-Tom[9]. Despite five independent transformation attempts, we were unable to generate null diploid *Slosd1* mutants (Supplementary Fig.3), which is consistent with a recent report that also failed to retrieve null mutants in the same gene[10] and suggests that *SlOSD1* has essential functions in mitotic division in tomato.

In contrast to *SlOSD1*, we isolated 33 T_0 diploid plants with the construct targeting *SlTAM*, and further characterized five different alleles (*Sltam-1* has the same mutation with *Sltam-6* in the inbred *MiMe* population) (Extended Data Fig. 1 and Supplementary Fig.4 and Supplementary Table 2). All five *Sltam* homozygous mutants exhibited normal vegetative development and visual inspection of pollen through scanning electron microscopy and Alexander staining indicated variable pollen sizes in *Sltam* homozygous mutants (Extended Data Fig.1b and Supplementary Fig.5, 6). Using high-throughput particle size detection, we quantified the diameter of pollen and found a monomodal size distribution in wild type pollen (98% reduced pollen), whereas all five *Sltam* lines produced pollen with a bimodal size distribution where 29-48% of pollen was unreduced (Extended Data Fig.1c, Supplementary Fig.6 and Supplementary Table 3). Cytological analysis of three *tam* alleles showed that many meiocytes stall at the dyad stage and do not enter meiosis II (Supplementary Figs.7-8). All five *Sltam* mutants produced lighter fruits that contained less seeds compared to wild-type controls (Supplementary Fig.9). Between 11-42% of seeds were undeveloped and abnormal in the five *Sltam* mutants whereas less than 1% of seeds were abnormal in wild type controls (Extended Data Fig.1d and Supplementary Fig.9). Despite the reduced seed production, all five *Sltam* mutants could produce seeds that were considerably larger than wild-type seeds which gave rise to tetraploid offspring (Extended Data Fig.1e, 1f and Supplementary Fig.6e). We also measured unreduced female gamete frequency by inference by performing crossing experiments using the *Sltam-3* and *Sltam-4* mutants we developed in the course of this study (Supplementary Fig.10). Wild type (MT) x wild type (MT) crosses resulted in normal seeds which could be cut open and rescued embryos gave rise to normal diploid plants (MT x MT, 48/48) (Supplementary Fig.10). In contrast, when we cut open the *tam* mutant crosses we already found many non-rescuable seeds (*Sltam-3* x MT, 25/153; *Sltam-4* x MT, 48/201) which we conclude are a result of failed endosperm development due to maternal excess (Supplementary Fig.10). We rescued the remaining embryos from the *Sltam* crosses and later found those embryos had three different

outcomes (viable & diploid; viable & triploid, non-viable & dead) (Supplementary Fig.10). We attribute the sizable “non-viable and dead” class to the triploid block, and conclude that even though the embryo was rescuable that it was likely already severely malnourished/not properly formed early in plant development. In summary, if we attribute all embryo outcomes that are not viable & diploid to the presence of an unreduced female gamete then we could estimate that in *Sltam*-3 (70/153) 46% and in *Sltam-4* (111/201) 55% of female gametes are unreduced. More cautiously, some of the failed embryo development could be due to reasons other than the triploid block so a conservative estimate may suggest a female unreduced gamete frequency of between 30-50%. This number would be consistent with estimates for unreduced male gametes where all five *tam* lines produced pollen where 29-48% of pollen was unreduced (Fig.1c). Further corroboration of these numbers is found from the ploidy of *Sltam* mutant selfing offspring. We found that from *Sltam-3* selfing seed we retrieved 5 tetraploid plants out of 73 offspring, and for *Sltam-4* we retrieved 7 tetraploid plants out of 66 offspring (Extended Data Fig.1f). This is a cumulative tetraploidy rate of 9.3% which could be explained by 30% male unreduced gametes and 30% female unreduced gametes (0.3*0.3*100 = 9%). Taken together, *Sltam* mutants can skip the second meiotic division at partial penetrance, in both male and female meiosis, leading to the generation of unreduced gametes.

To further confirm the conserved meiotic function of SPORULATION 11-1 (SPO11-1) and REC8 in tomato, we obtained two independent alleles of *Slspo11-1* and *Slrec8* mutants in Micro-Tom background (Supplementary Table 2, Supplementary Fig.11 and Supplementary Fig.12). To explore the reasons for complete male sterility in both single mutants (Extended Data Fig.2b and Supplementary Fig.13), we carried out observations of chromosome behavior in male meiocytes. In *Slspo11-1-1* mutants, an abnormal meiotic process was observed where twenty–four univalent were present instead of twelve bivalents at diakinesis, indicating no meiotic recombination (Supplementary Fig.14). The random segregation of homologous chromatids during meiosis I and subsequent segregation of sister chromatids during meiosis II resulted in aborted polyads (Supplementary Fig.14). In *Slrec8-1* mutants, there is no typical pachytene stage due to defective homologous chromosome pairing (Supplementary Fig.15). At diakinesis, abnormal tangled chromosomes with partial chromosome bridges occurred. Subsequently we observed more than 12 chromosomes randomly segregating towards the two opposite poles at anaphase I, indicating premature separation of sister chromatids at meiosis I (Supplementary Fig.15). In *Slspo11-1 Slrec8* double mutants, the first meiotic division mimics the mitotic cell division leading to the balanced segregation of sister chromatids and then the second division is unbalanced resulting in aneuploid gametes (Supplementary Fig.16).

Supplementary Note 2. A chromosome-scale *de novo* genome assembly of Micro-Tom.

A chromosome-scale *de novo* genome assembly of the dwarf tomato variety Micro-Tom was generated using PacBio HiFi reads and scaffolded using chromosome confirmation capture. We generated PacBio HiFi reads from Micro-Tom using two different insert sizes leading to a total of 47.3 Gbp raw data. Micro-Tom is a highly inbred tomato variety which is expected to have very low heterozygosity. This was confirmed by k-mer analysis (k=21) of the HiFi data which revealed only a single clear peak (Supplementary Fig.22a), and therefore the genome was assembled as a single haplotype. The HiFi data was assembled using Hifiasm[11], resulting in a total length of 925Mbp with an N50 value of 21.1 Mbp (Supplementary Table 5).

Next, we generated 41.5 Gbp of chromosome confirmation capture data (Omni-C, Dovetail) and used it for automated scaffolding using Salsa[12]. Contiguity of the assembly was further

improved through multiple rounds of manual scaffolding and scaffolding correction, until a chromosome-scale genome was obtained (Supplementary Fig.22b). The final assembly was well supported by chromatin confirmation capture interactions (Supplementary Fig.22b). Dotplot comparison of the scaffolded Micro-Tom genome to the Moneyberg-TMV (MbTMV) genome[13] showed a highly co-linear alignment, except for chromosome 9, which contains a 64.1 Mbp introgression from *S. peruvianum* in MbTMV (Supplementary Fig.22c). Unplaced Micro-Tom sequences aligned to Moneyberg-TMV "ch00"[13] (Supplementary Fig.22c) which is dense in chloroplast, mitochondrial, rDNA and satellite derived repeats, and did not show any OmniC interaction with any of the scaffolded chromosomes (Supplementary Fig.22b).

Next we took several measures to assess the quality of the Micro-Tom genome assembly. K-mer based assembly evaluation using Merqury[14] showed the Micro-Tom genome assembly was 99.22% complete (QV=72.40) (Supplementary Table 6). The assembly was further validated by means of read coverage analysis and characterization of nucleotide composition and repeat elements (SSR and LTR) (Supplementary Fig.23). We found coverage was mostly even along the twelve chromosomes, and in most cases regions with extended reduced coverage overlaid drastic changes in nucleotide composition. These regions largely overlap with tandem arrays of repeat sequences including 45S rDNA intergenic spacer (IGS), 45S rDNA (complete sequence), telomeric repeat or Tomato genomic repeat 1 (TGR1). To check gene completeness, BUSCO (Benchmarking Universal Single-Copy Orthologs) analysis was performed using the Solanales gene set. We found 98.3% (5851/5950) of expected genes were present in a complete form[15], while 12/5950 were fragmented and 87/5950 were absent (Supplementary Table 6). The assembly of repeat content was checked using the LTR assembly index (LAI=14.05)[16] (Supplementary Table 6). Overall, our results suggest our assembly of the Micro-Tom genome is near-complete and of high-quality.

Supplementary Note References:

1. Mieulet, D. *et al.* Turning rice meiosis into mitosis. *Cell Res.* **26**, 1242–1254 (2016).
2. d'Erfurth, I. *et al.* Turning Meiosis into Mitosis. *PLoS Biol.* **7**, e1000124 (2009).
3. Wang, Y., Van Rengs, W. M. J., Zaidan, M. W. A. M. & Underwood, C. J. Meiosis in crops: From genes to genomes. *J. Exp. Bot.* **72**, 6091–6109 (2021).
4. Iwata, E. *et al.* GIGAS CELL1, a novel negative regulator of the anaphase-promoting complex/cyclosome, is required for proper mitotic progression and cell fate determination in Arabidopsis. *Plant Cell* **23**, 4382–4393 (2011).
5. Underwood, C. J. & Mercier, R. Engineering Apomixis: Clonal Seeds Approaching the Fields. *Annu. Rev. Plant Biol.* **73**, 201–225 (2022).
6. d'Erfurth, I. *et al.* The CYCLIN-A CYCA1;2/TAM Is Required for the Meiosis I to Meiosis II Transition and Cooperates with OSD1 for the Prophase to First Meiotic Division Transition. *PLoS Genet.* **6**, e1000989 (2010).
7. Cifuentes, M. *et al.* TDM1 Regulation Determines the Number of Meiotic Divisions. *PLoS Genet.* **12**, (2016).
8. Magnard, J. L., Yang, M., Chen, Y. C. S., Leary, M. & McCormick, S. The Arabidopsis Gene Tardy Asynchronous Meiosis Is Required for the Normal Pace and Synchrony of Cell Division during Male Meiosis. *Plant Physiol.* **127**, 1157–1166 (2001).
9. SCOTT, J. W. & HARBAUGH, B. K. Micro-tom. A miniature dwarf tomato. *Circ. Univ. Florida. Agric. Exp. Station.* (1989).
10. Di, S. *et al.* Tomato UVI4 homologue modulates cell expansion to participate heat-stimulated hypocotyl elongation. *Environ. Exp. Bot.* **201**, 104963 (2022).
11. Cheng, H., Concepcion, G. T., Feng, X., Zhang, H. & Li, H. Haplotype-resolved de novo assembly using phased assembly graphs with hifiasm. *Nat. Methods* **18**, 170–175 (2021).
12. Ghurye, J. *et al.* Integrating Hi-C links with assembly graphs for chromosome-scale assembly. *PLOS Comput. Biol.* **15**, e1007273 (2019).
13. van Rengs, W. M. J. *et al.* A chromosome scale tomato genome built from complementary PacBio and Nanopore sequences alone reveals extensive linkage drag during breeding. *Plant J.* **110**, 572–588 (2022).
14. Rhie, A., Walenz, B. P., Koren, S. & Phillippy, A. M. Merqury: Reference-free quality, completeness, and phasing assessment for genome assemblies. *Genome Biol.* **21**, 1–27 (2020).
15. Simão, F. A., Waterhouse, R. M., Ioannidis, P., Kriventseva, E. V. & Zdobnov, E. M. BUSCO: Assessing genome assembly and annotation completeness with single-copy orthologs. *Bioinformatics* **31**, 3210–3212 (2015).
16. Ou, S., Chen, J. & Jiang, N. Assessing genome assembly quality using the LTR Assembly Index (LAI). *Nucleic Acids Res.* **46**, e126 (2018).

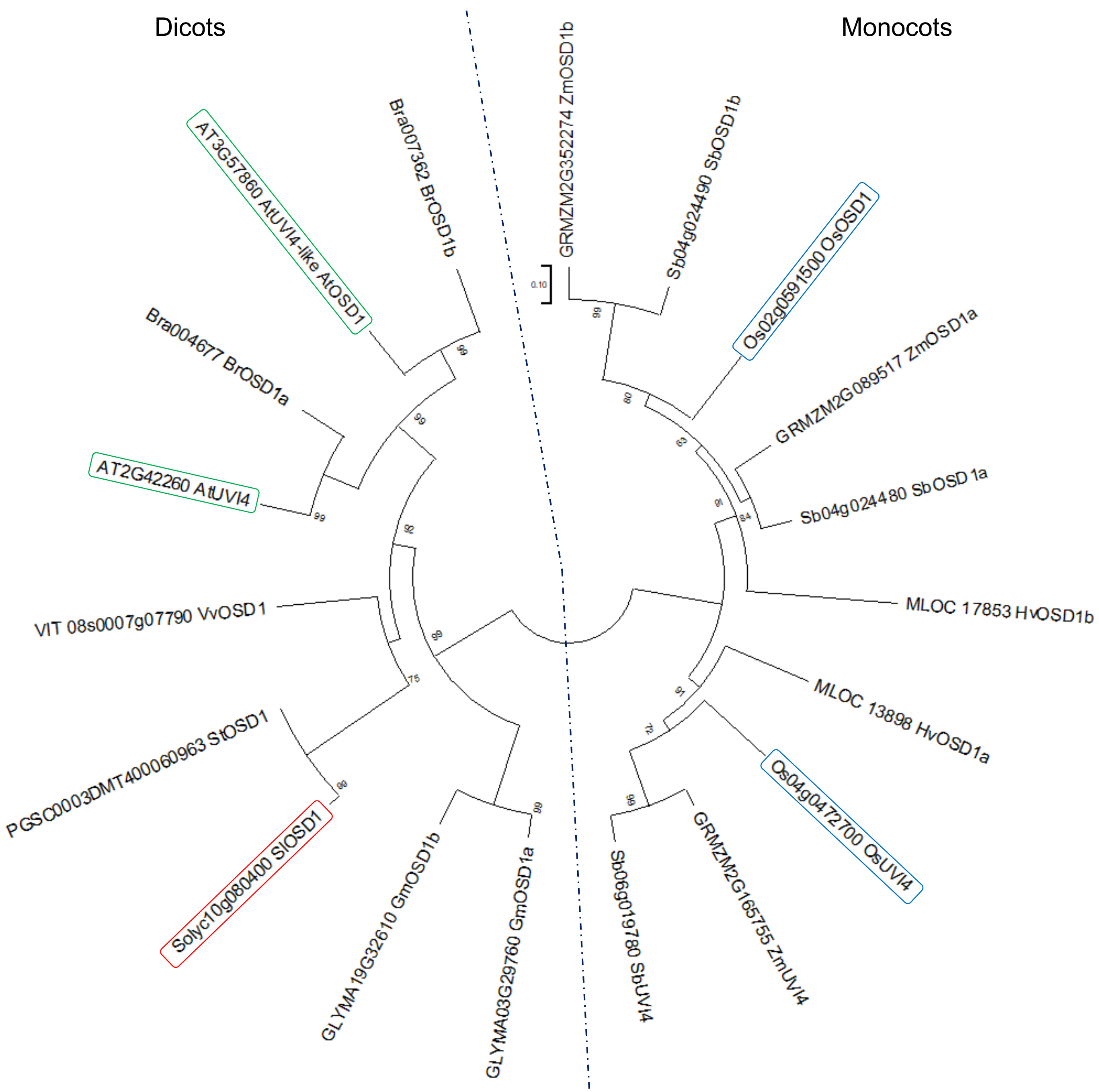

Supplementary Figure 1: A phylogenetic tree of OSD1 in selected monocot and dicot plant species.

OSD1 protein sequences from thale cress (*Arabidopsis thaliana*), Chinese cabbage (*Brassica rapa*), soybean (*Glycine max*), barley (*Hordeum vulgare*), rice (*Oryza sativa*) tomato (*Solanum lycopersicum*), potato (*Solanum tuberosum*), sorghum (*Sorghum bicolor*), grape (*Vitis vinifera*) and maize (*Zea mays*) were extracted from NCBI genomic databases. BLAST of the protein sequences was carried out using clustalX2.1 software and the phylogenetic tree was generated through MEGA11 software. For OSD1/UVI4, there is only single copy in tomato (red box), whereas in Arabidopsis (green boxes) and rice (blue boxes) there are two genes.

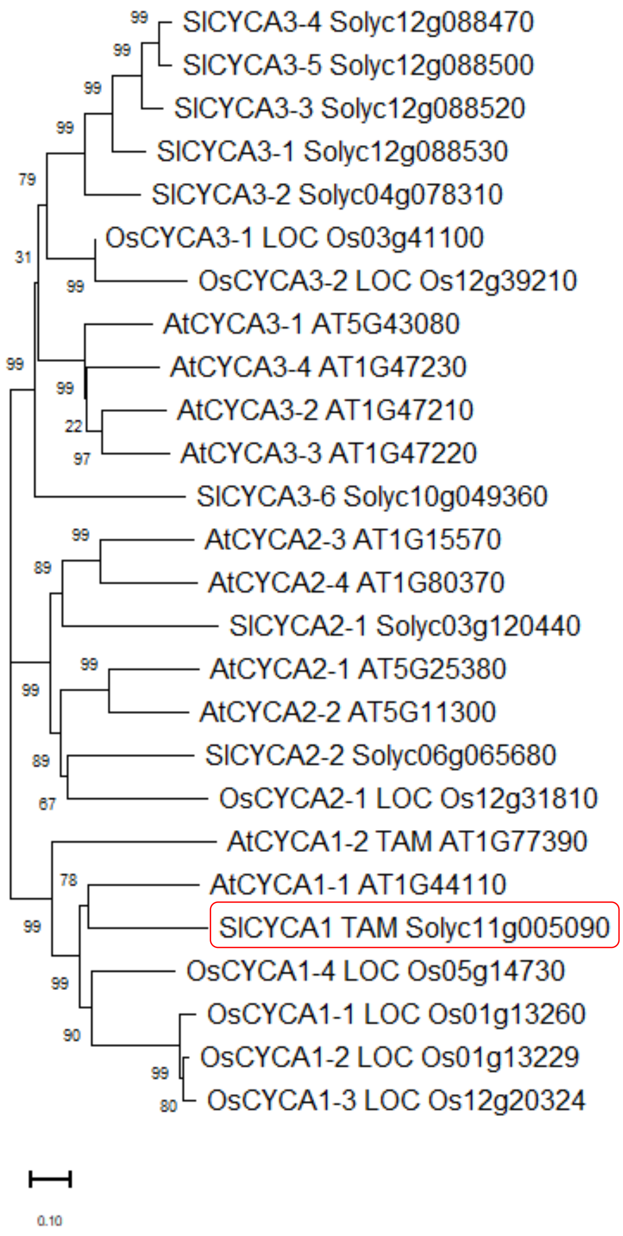

Supplementary Figure 2: A phylogenetic tree of cyclin A proteins from Arabidopsis, rice and tomato.

Cyclin A protein sequences from Arabidopsis, rice and tomato were extracted from NCBI genomic databases. BLAST of the protein sequences was carried out using clustalX2.1 software and the phylogenetic tree was generated through MEGA11 software. For cyclinA1, there is only single copy in tomato (red box).

a

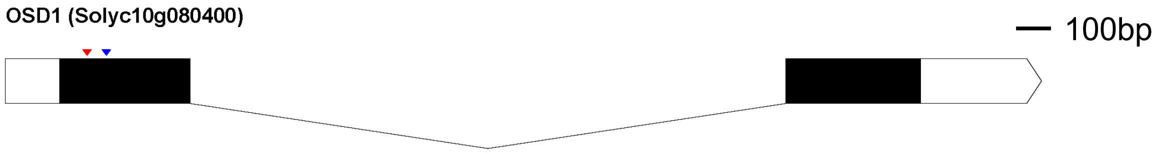

sgRNA1: AGGTGGCATTGAGATCTTCGAGG
sgRNA2: TACAGACTACAGCAGAAGCAGGG

b

pYZ178_1

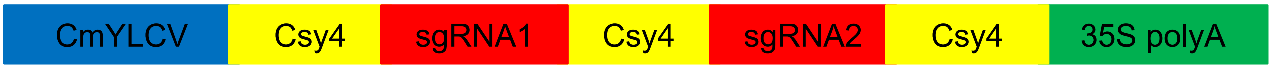

Transformation of plasmid pYZ178_1 (with two sgRNAs targeting the first exon of Solyc10g080400) into Micro-Tom was carried out 3 times. For each transformation more than 100 explants were infected leading to 0, 0 and 1 regenerated transgenic plants for each transformation whereas comparable transformations that were carried out in parallel were able to generate at least 10 transgenic plants per transformation. The one transgenic plant regenerated with this construct was tetraploid and had frameshift mutations on 2 of 4 alleles with the following genotype:

Allele 1: TACAGACTACAGCAGAAGCAGGG……………………48%
Allele 2: TACAGACTACAGCAGA-GCAGGG (-1 bp)……………26%
Allele 3: TACAGACTACAGCAGAAAGCAGGG (+1 bp)………..21%

pYZ178_2

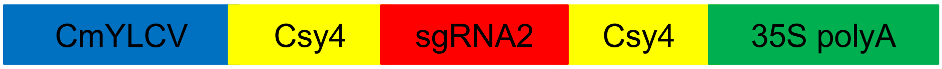

Transformation of plasmid pYZ178_2 (with one sgRNA targeting the first exon of Solyc10g080400) into Micro-Tom was carried out 2 times. For each transformation more than 100 explants were infected leading to 1 and 3 plants in each transformation whereas comparable transformations that were carried out in parallel were able to generate at least 10 transgenic plants per transformation. All four plants containing pYZ178_2 were tetraploid and none contained predicted null alleles on all haplotypes. An example genotype of one of the four plants was:

Allele 1: TACAGACTACAGCAGAAGCAGGG……………………51%
Allele 2: TACAGACTAC-------GCAGGG (-7 bp)…………………16%
Allele 3: T-----------------------------------CCAG (-35 bp)…………….12%

c

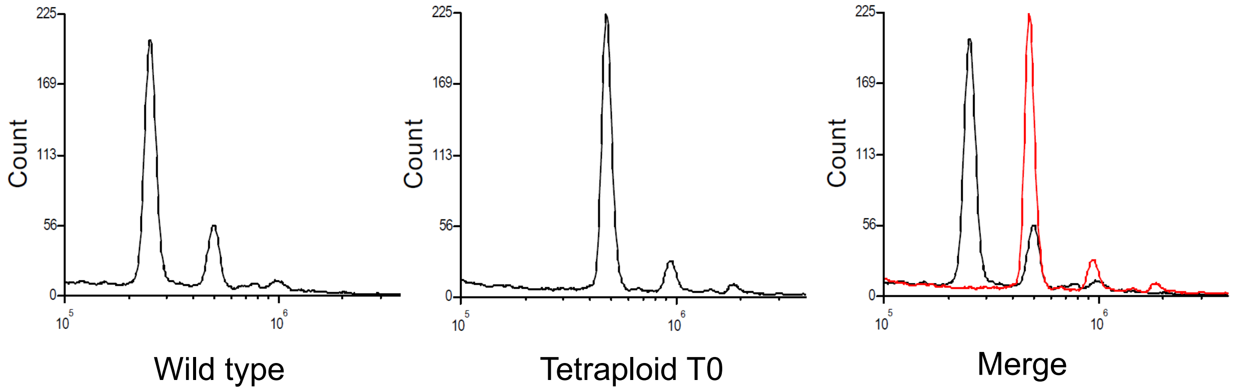

Supplementary Figure 3: SlOSD1/SlUVI4 transformation in inbred tomato.

a, Schematic representation of Solyc10g080400 (*SlOSD1/UVI4*) gene structure in tomato. The gene structure is based on the ITAG release 4.0 from Sol Genomics. The filled black boxes represent exons and the empty boxes represent the 5' UTR and 3' UTR. Red and blue solid inverted triangles are two different guide RNAs. Scale bar=100bp.

b, Overview of two different CRISPR/Cas9 mutagenesis vectors were created that target SlOSD1/UVI4 in tomato. using two or one sgRNAs in tomato. The tetraploid offsprings showed different percentages of mutations.

c, Flow cytometry results of wild type and a tetraploid T0 plant containing pYZ178_2. Left (diploid wild type), middle (tetraploid T0 plant) and right (the merged result where the tetraploid plant is depicted in red).

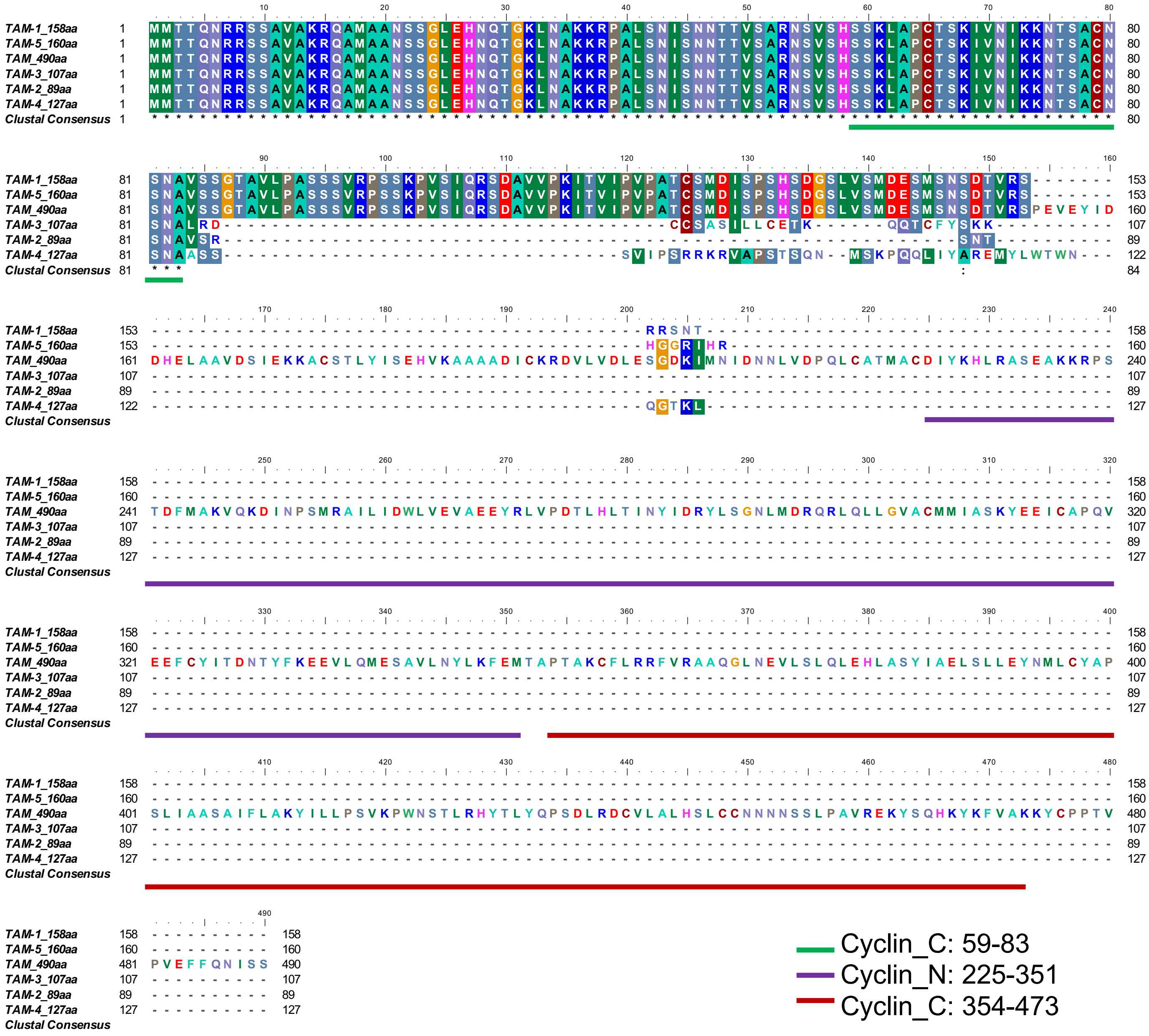

Supplementary Figure 4: Protein sequence alignment of SlTAM with the sequences of five different *Sltam* alleles. Alignment of the amino acid sequence of SlTAM protein with three conserved domains (one Cyclin_N and two Cyclin_Cs) together with 5 different *tam* alleles presented in Fig 1. The multiple protein sequences were aligned by BLAST through clustalX2. The conserved domains were identified using the Pfam protein database (http://pfam.xfam.org/). The mutation in the *tam-6* allele is identical to the *tam-1* allele.

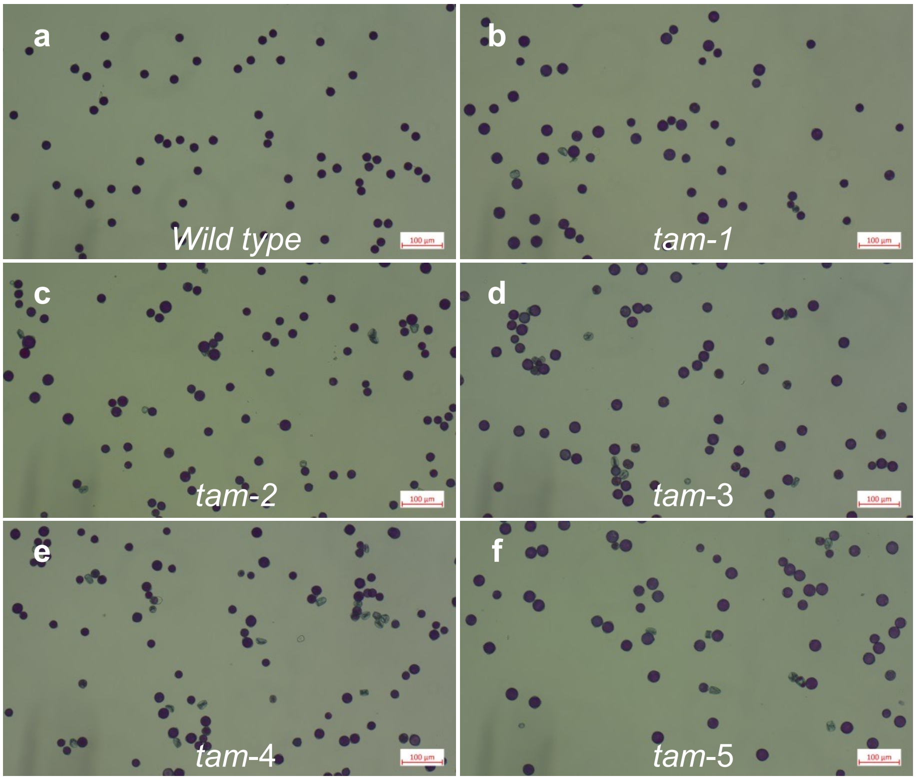

Supplementary Figure 5: Alexander staining results of pollen from wild type Micro-Tom and five different *Sltam* mutants.

Alexander staining results of pollen from wild type Micro-Tom (n= 37) (**a**) and five different *Sltam* mutants (**b**-**f**) (*tam-1*, n= 28; *tam-2*, n= 24; *tam-3*, n= 27; *tam-4*, n= 25; *tam-5*, n= 36). Darkly staining pollen is viable and non-colored pollen is non-viable. Scale bar = 100 μm.

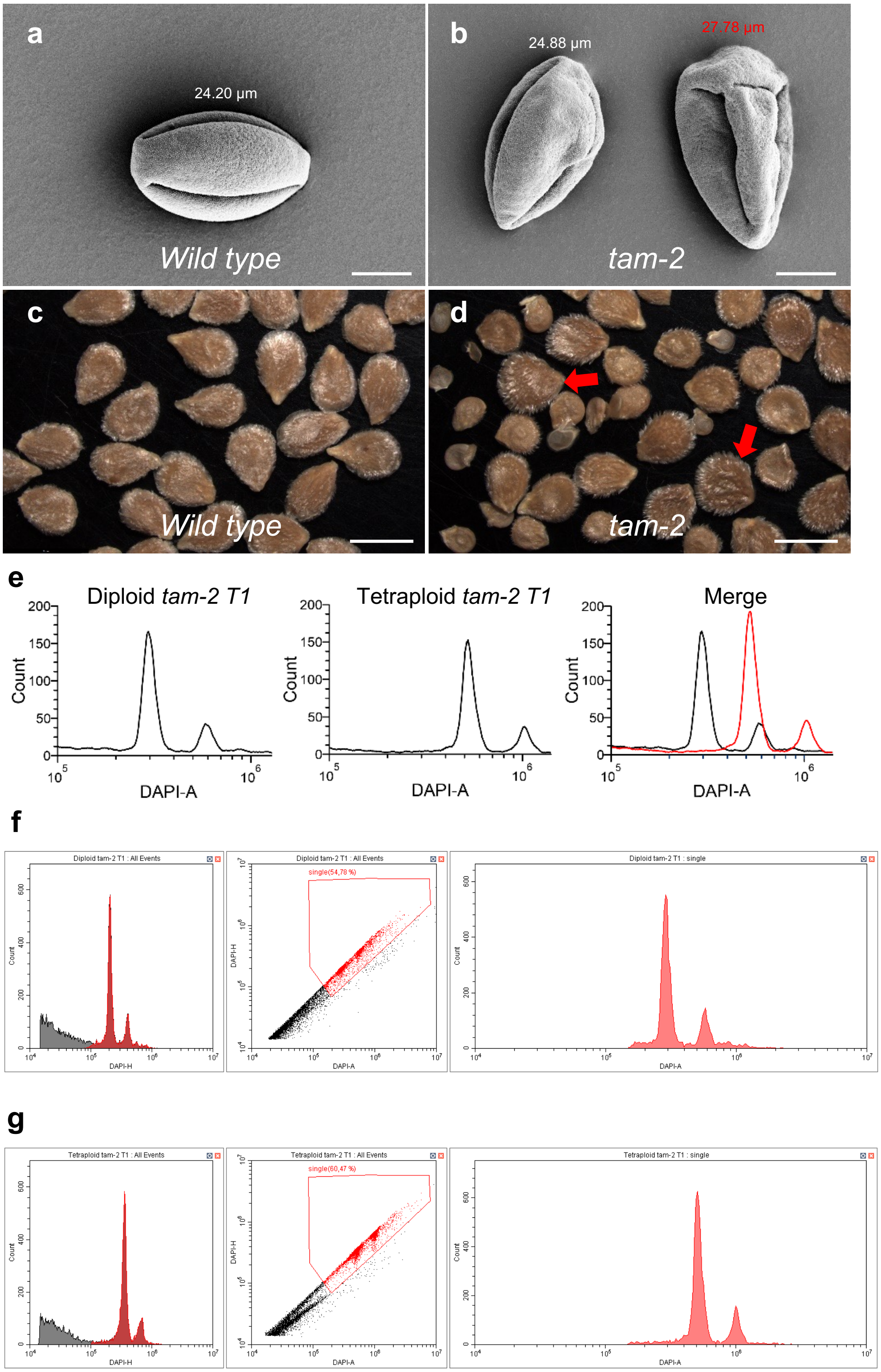

Supplementary Figure 6: SEM pollen data, selfed seeds and ploidy levels of wild type Micro-Tom and the *Sltam-2* mutant.

Scanning electron microscope (SEM) images of wild type Micro-Tom pollen (n= 50) (**a**) and *tam-2* pollen (n= 42) (**b**), Scale bar = 10 µm. Images of selfing seeds from wild type Micro-Tom (n= 21) (**c**) and *Sltam-2* mutant (n= 23) (**d**), Scale bar = 3 mm. The red arrows highlight bigger seeds that give rise to tetraploid offspring. **e**, Flow cytometry result of diploid *Sltam-2* T1 single mutant (left), tetraploid *Sltam-2* T1 single mutant (middle) and the merged result (right) where the tetraploid plant is depicted in red. **f**, Flow cytometry results before gating (left), gating strategy (middle) and flow cytometry results after gating (right) in the diploid *Sltam-2* T1 single mutant. **g**, Flow cytometry results before gating (left), gating strategy (middle) and flow cytometry results after gating (right) in the tetraploid *Sltam-2* T1 single mutant.

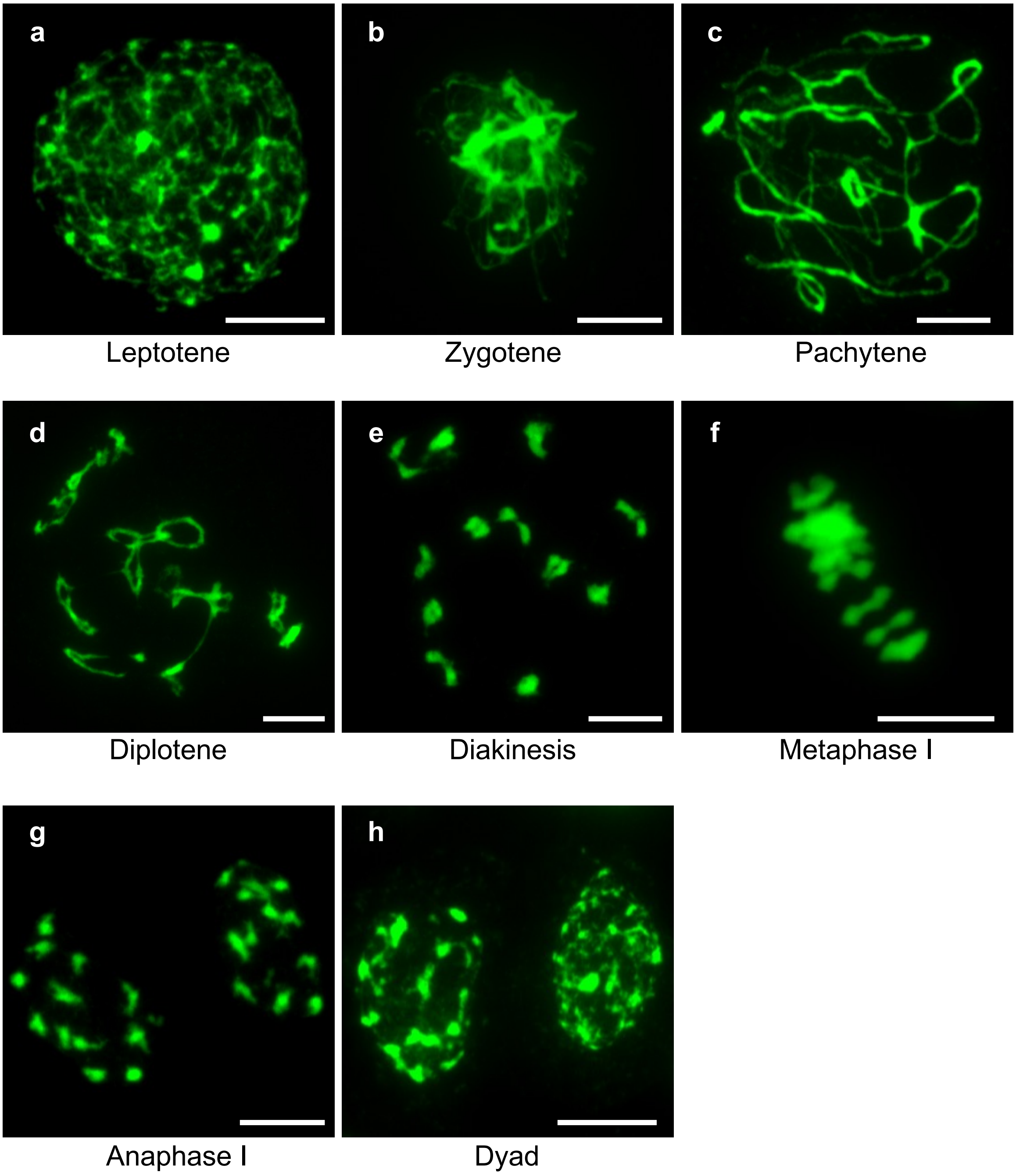

Supplementary Figure 7: Meiotic chromosome behaviour in the *Sltam-1* mutant.
Chromosome behaviour during male meiosis in the *Sltam-1* mutant in the Micro-Tom inbred line.
a, Leptotene (n= 21); **b**, Zygotene (n= 54); **c**, Pachytene (n= 43); **d**, Diplotene (n= 39); **e**, Diakinesis (n= 53); **f**, Metaphase I (n= 26); **g**, Anaphase I (n= 42); **h**, Dyad (n= 36). Scale bars = 10 µm.

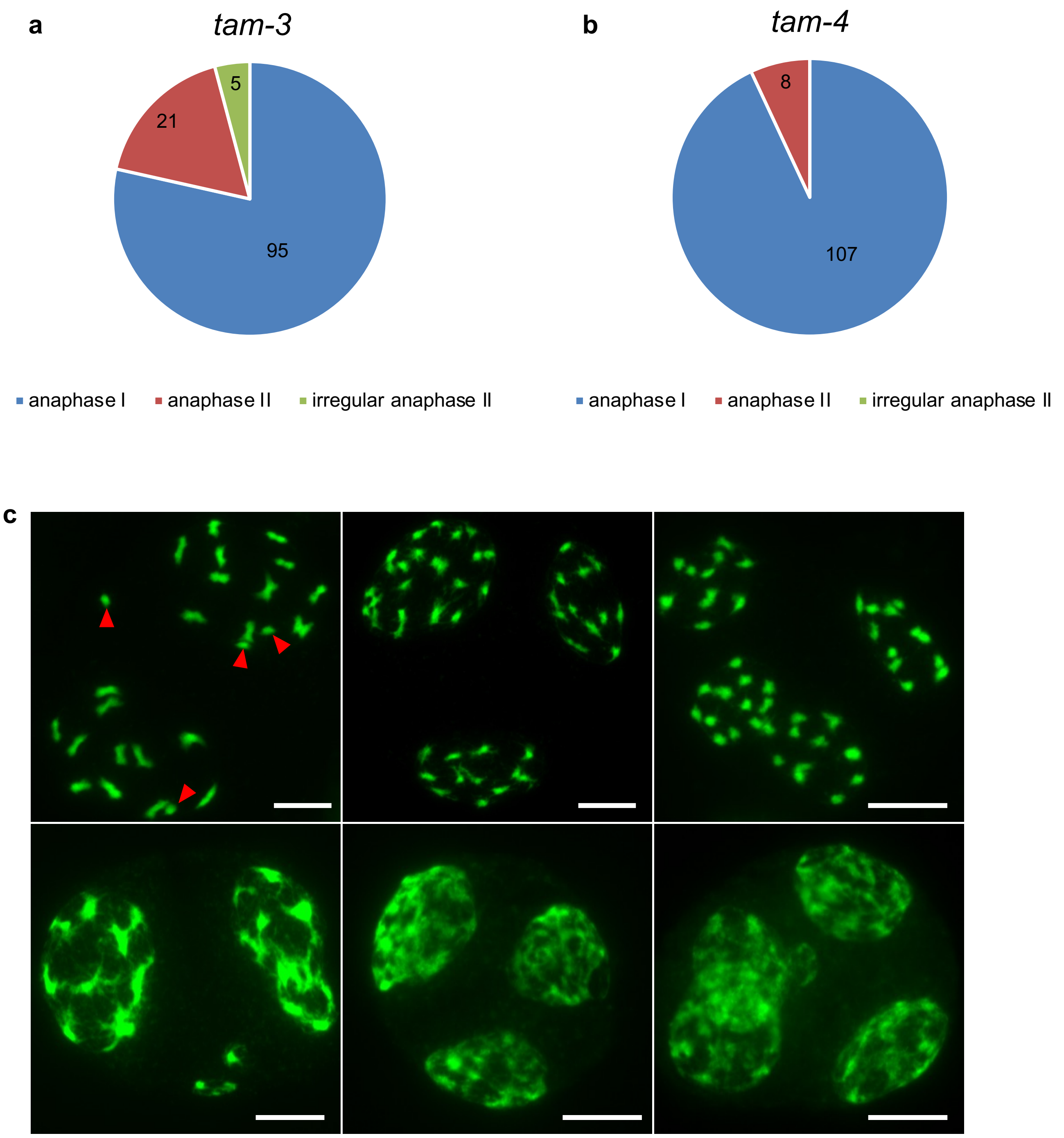

Supplementary Figure 8: Quantification of meiosis progression in *Sltam* mutants and examples of abnormal meiotic behaviour.
a, Quantification of meiotic stages within single slides of *Sltam-3.* This result indicates up to 78% of meiotic cells could exit the meiotic cell cycle after meiosis I.
b, Quantification of meiotic stages within single slides of *Sltam-4.* This result indicates up to 93% of meiotic cells could exit the meiotic cell cycle after meiosis I.
c, Examples of abnormal chromosome behaviour observed in *Sltam-3* mutant meiocytes. From top left to top right; Anaphase I, Anaphase II, Telophase II. From bottom left to bottom right; Dyad, Triad, Polyad. Scale bar = 10 µm.

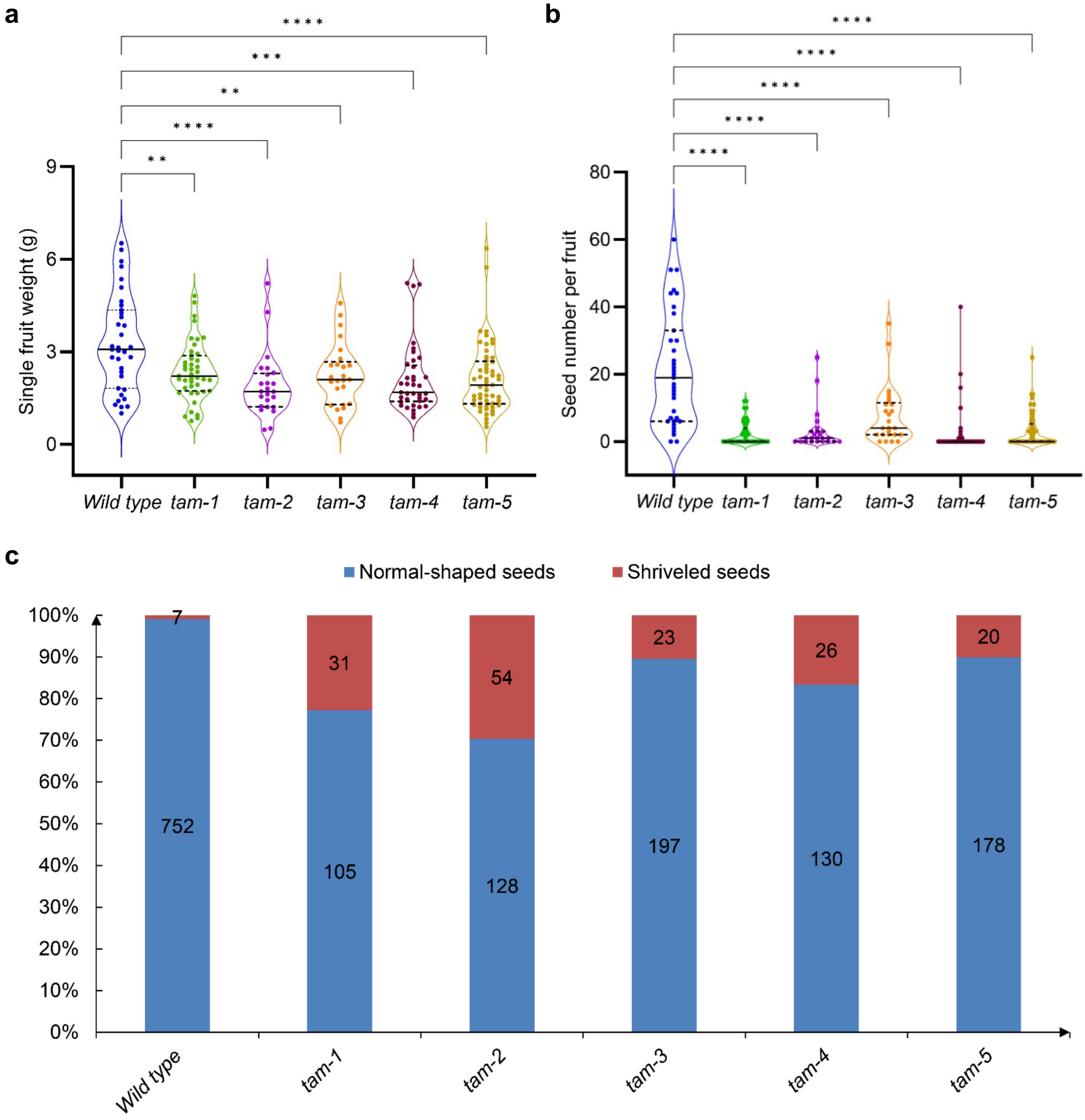

Supplementary Figure 9: Analysis of fruit weight and seed number per fruit in wild type and *Sltam* mutants.

a, Differences in fruit weights were analysed using the ordinary one-way ANOVA. Šídák's multiple comparisons test was used to determine significant differences between the *tam* mutants and wild type. Wild type (n=35), *tam-1* (n=43, P=0.0032), *tam-2* (n=24, P=8.90×10^{-5}), *tam-3* (n=25, P=0.0037), *tam-4* (n=37, P= 0.0005) and *tam-5* (n=54, P=5.17×10^{-5}).

b, Differences in seed number per fruit were analysed using the ordinary one-way ANOVA. Šídák's multiple comparisons test was used to determine significant differences between the *tam* mutants and wild type. Wild type (n=35), *tam-1* (n=43, P=8.35×10^{-8}), *tam-2* (n=24, P=4.05×10^{-7}), *tam-3* (n=25, P=6.99×10^{-5}), *tam-4* (n=37, P=1.91×10^{-7}) and *tam-5* (n=54, P=2.47×10^{-7}).

c, Analysis of selfed seeds collected from wild type and *Sltam* mutants. Seeds were visually classified as normal-shaped (fully developed in appearance) or shriveled (not fully developed and shriveled often just an empty seed coat). The fruit numbers that were analysed for each genotype is as follows: Wild type (n=35), *tam-1* (n=43), *tam-2* (n=24), *tam-3* (n=25), *tam-4* (n=37) and *tam-5* (n=54).

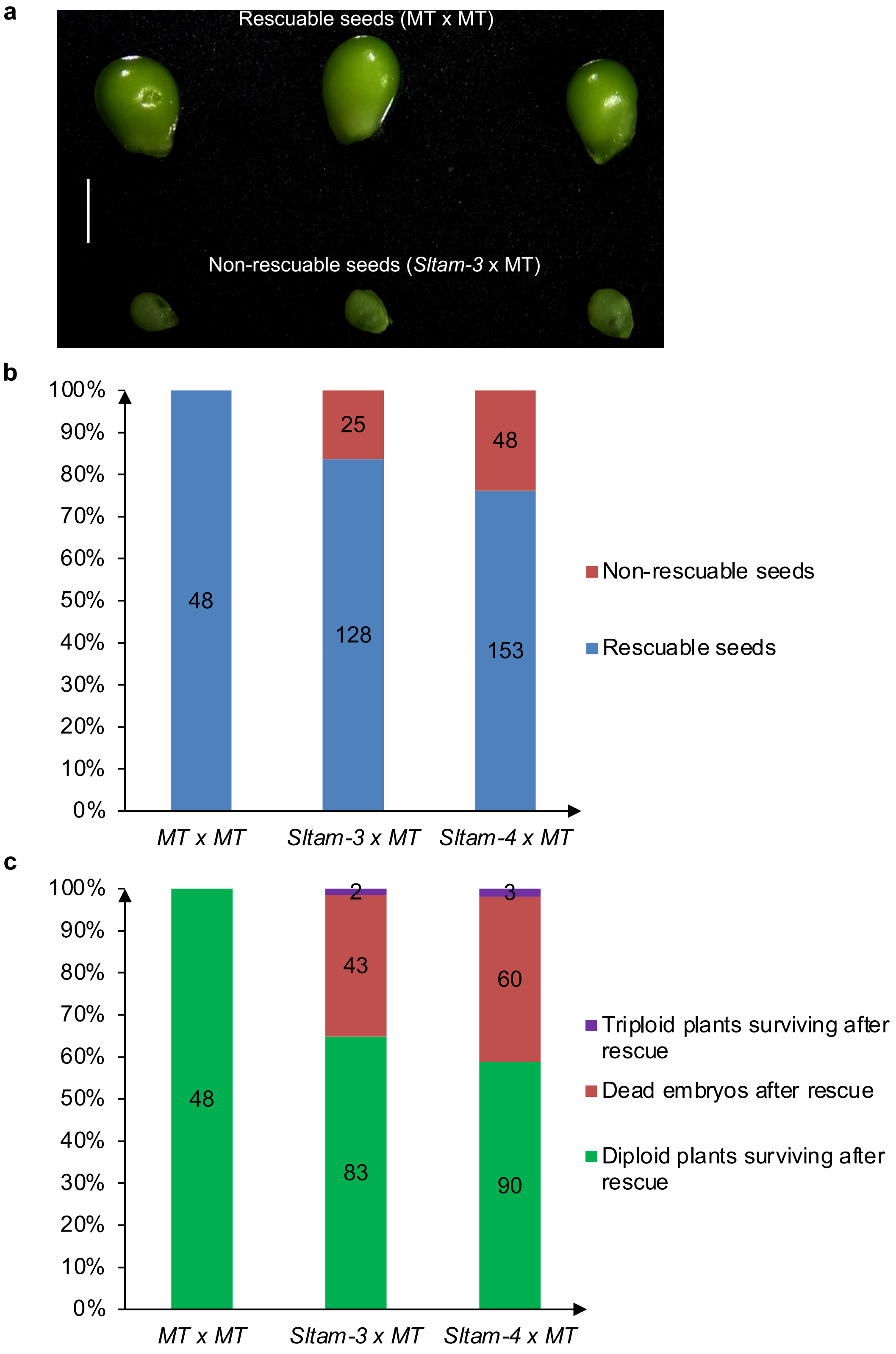

Supplementary Figure 10: Embryo rescue from developing seeds after pollination of *Sltam* mutants with wild type pollen.
a, Rescuable and non-rescuable seeds found in Micro-Tom and *Sltam* mutant fruits after manual polination with wild type pollen from Micro-Tom (MT). The fruits were opened 21 days after pollination. Scale bar = 1mm.
b, Quantitative analysis of rescuable and non-rescuable seeds in crossed fruits for wild type (n=48) and *Sltam* (*Sltam-3*, n=153; *Sltam-4*, n=201).
c, Quantitative analysis of the outcome of rescued embryos in wild type (n=48) and *Sltam* (*Sltam-3*, n=128; *Sltam-4*, n=153).

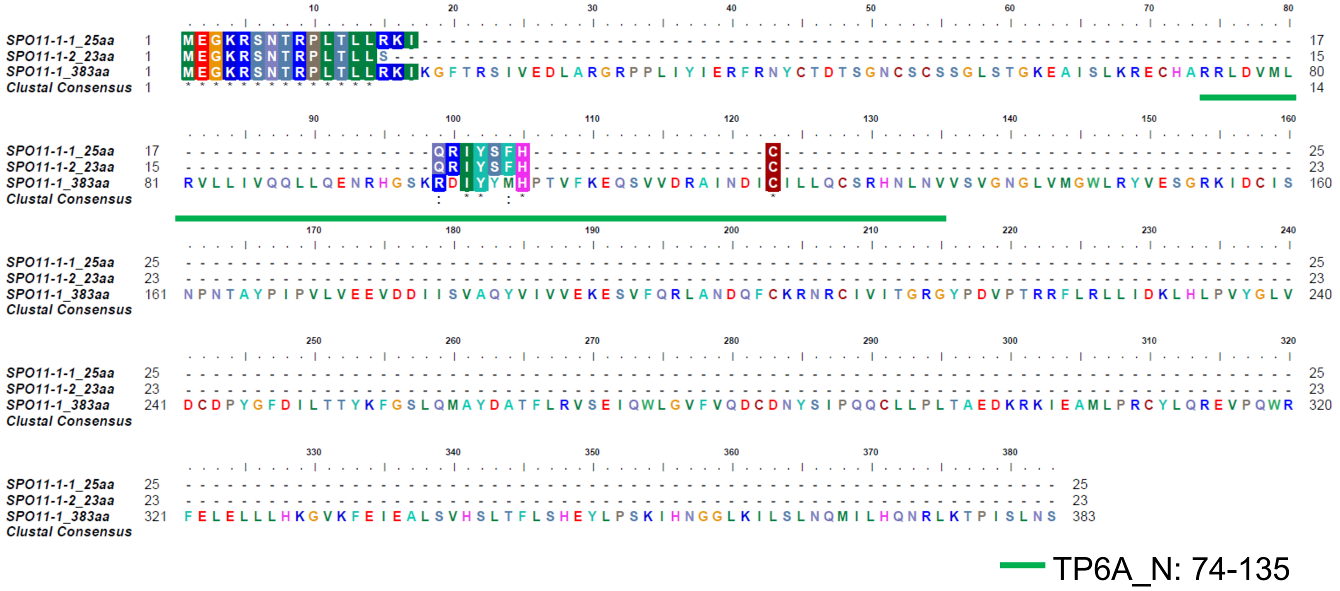

Supplementary Figure 11: Protein sequence alignment of SlSPO11-1 with the sequences of two different *Slspo11-1* alleles.

Alignment of the amino acid sequence of SlSPO11-1 protein with one conserved domain (TP6A_N) together with two different alleles. The multiple protein sequences were aligned by BLAST through clustalX2. The conserved domains were identified using the Pfam protein database (http://pfam.xfam.org/).

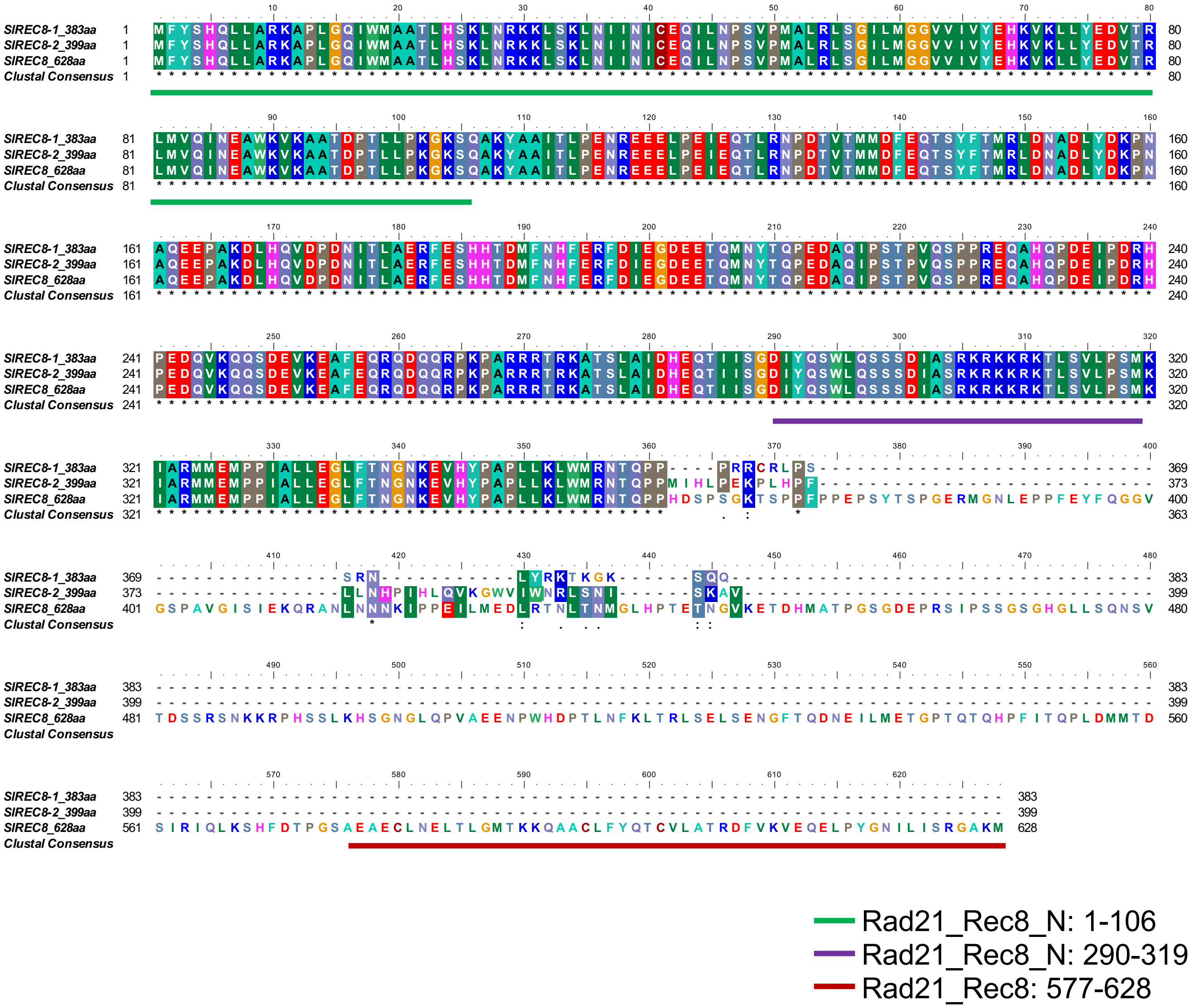

Supplementary Figure 12: Protein sequence alignment of SlREC8 with the sequences of two different *Slrec8* alleles.

Alignment of the amino acid sequence of SlREC8 protein with three conserved domains together with two different *Slrec8* allele sequences. The multiple protein sequences were aligned by BLAST through clustalX2. The conserved domains were identified using the Pfam protein database (http://pfam.xfam.org/).

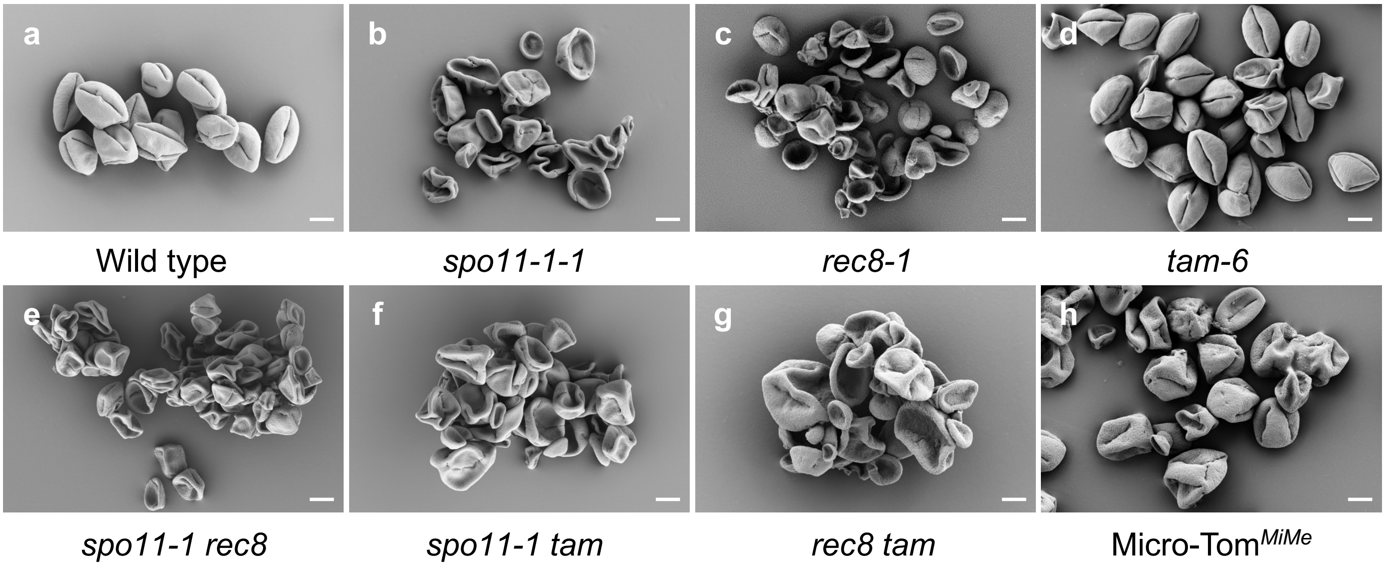

Supplementary Figure 13: Scanning electron microscopy (SEM) micrographs of pollen grains from wild type and mutant plants.

Scanning electron microscopy (SEM) micrographs of pollen grains from wild type (**a**, n=74), *Slspo11-1-1* (**b**, n=52), *Slrec8-1* (**c**, n=69), *Sltam-6* (**d**, n=73), *spo11-1 rec8* (**e**, n=56), *spo11-1 tam* (**f**, n=51)*, rec8 tam* (**g**, n=40) and Micro-Tom^{MiMe} (**h**, n=56) mutants. Scale bar = 10 µm.

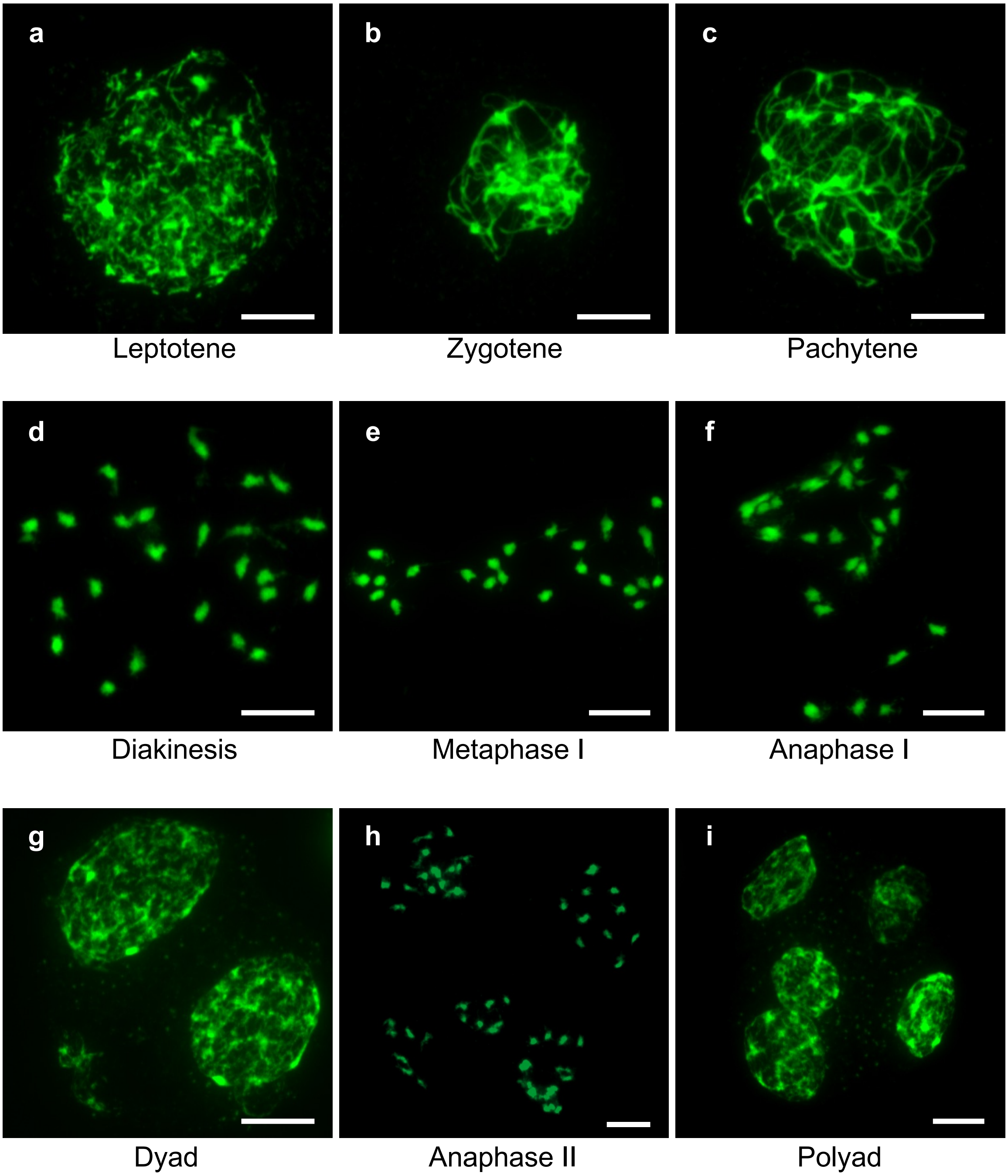

Supplementary Figure 14: Chromosome behaviour of male meiocytes in the *Slspo11-1-1* mutant in Micro-Tom.
a, Leptotene (n=23); **b**, Zygotene (n=42); **c**, Pachytene (n=26); **d**, Diakinesis (n=20); **e**, Metaphase I (n=24); **f**, Anaphase I (n=18); **g**, Dyad (n=22); **h**, Anaphase II (n=38); **i**, Polyad (n=43). Scale bar = 10 μm.

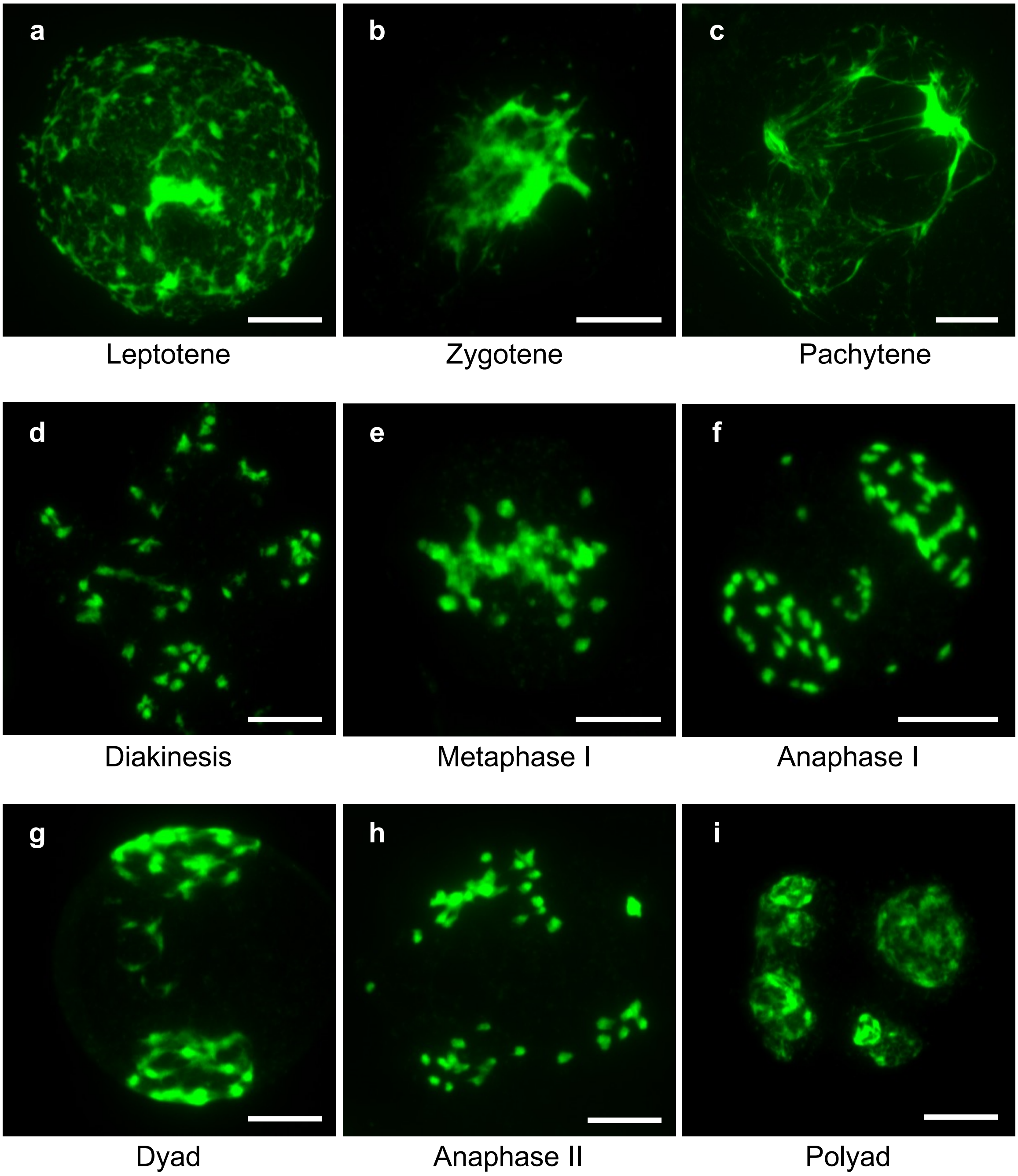

Supplementary Figure 15: Chromosome behaviour of male meiocytes in the *Slrec8-1* mutant in Micro-Tom.
a, Leptotene (n=23); **b**, Zygotene (n=41); **c**, Pachytene (n=23); **d**, Diakinesis (n=26); **e**, Metaphase I (n=18); **f**, Anaphase I (n=28); **g**, Dyad (n=17); **h**, Anaphase II (n=23); **i**, Polyad (n=42). Scale bar = 10 μm.

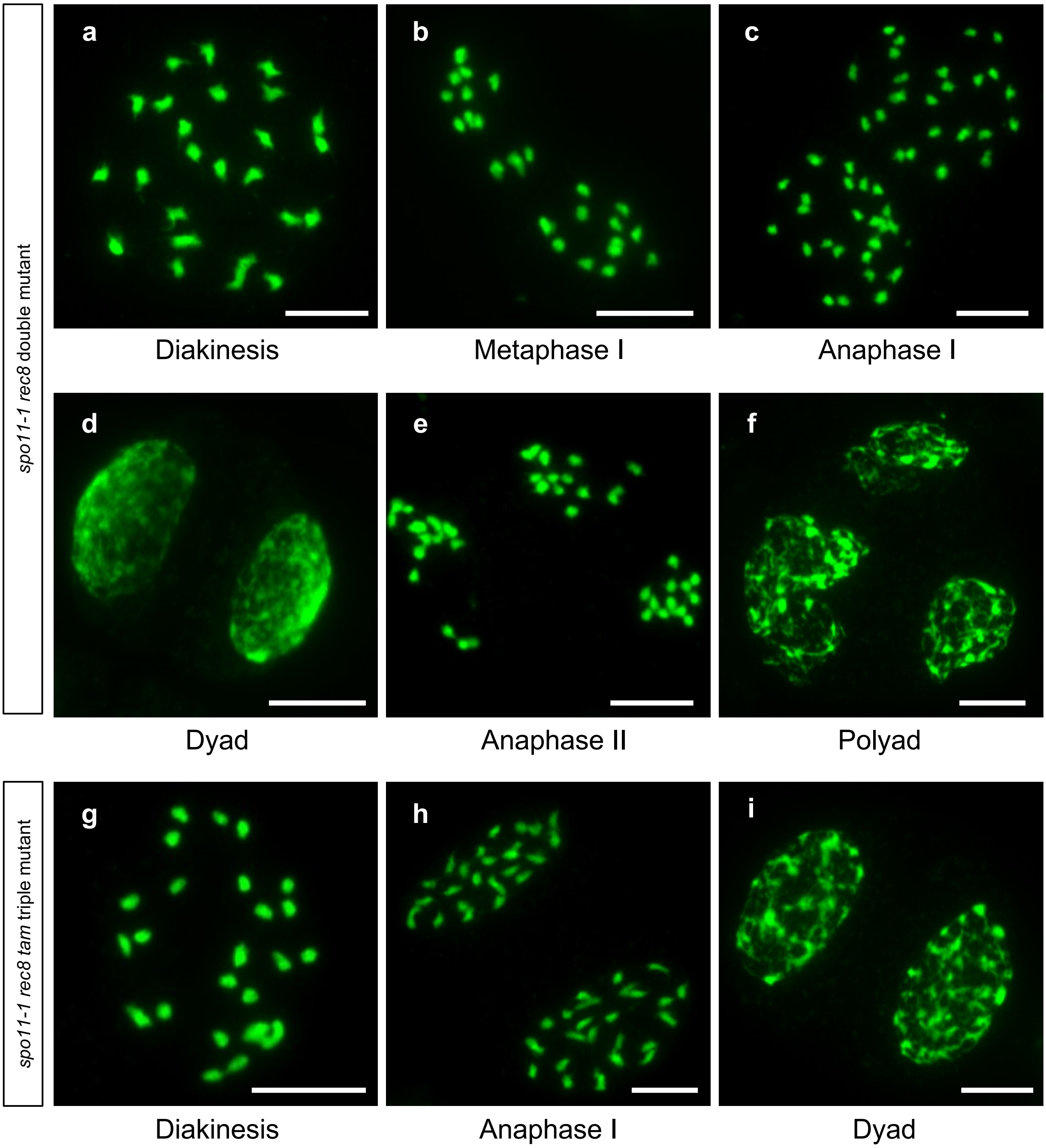

Supplementary Figure 16: Chromosome behaviour of male meiocytes in *spo11-1 rec8* and *spo11-1 rec8 tam* (Micro-Tom^{MiMe}) mutants in Micro-Tom.
Slspo11-1 rec8: **a**, Diakinesis (n=32); **b**, Metaphase I (n=25); **c**, Anaphase I (n=23); **d**, Dyad (n=31); **e**, Anaphase II (n=29); **f**, Polyad (n=43); *Slspo11-1 rec8 tam*: **g**, Diakinesis (n=36); **h**, Anaphase I (n=27); **i,** Dyad (n=38). Scale bar = 10 µm.

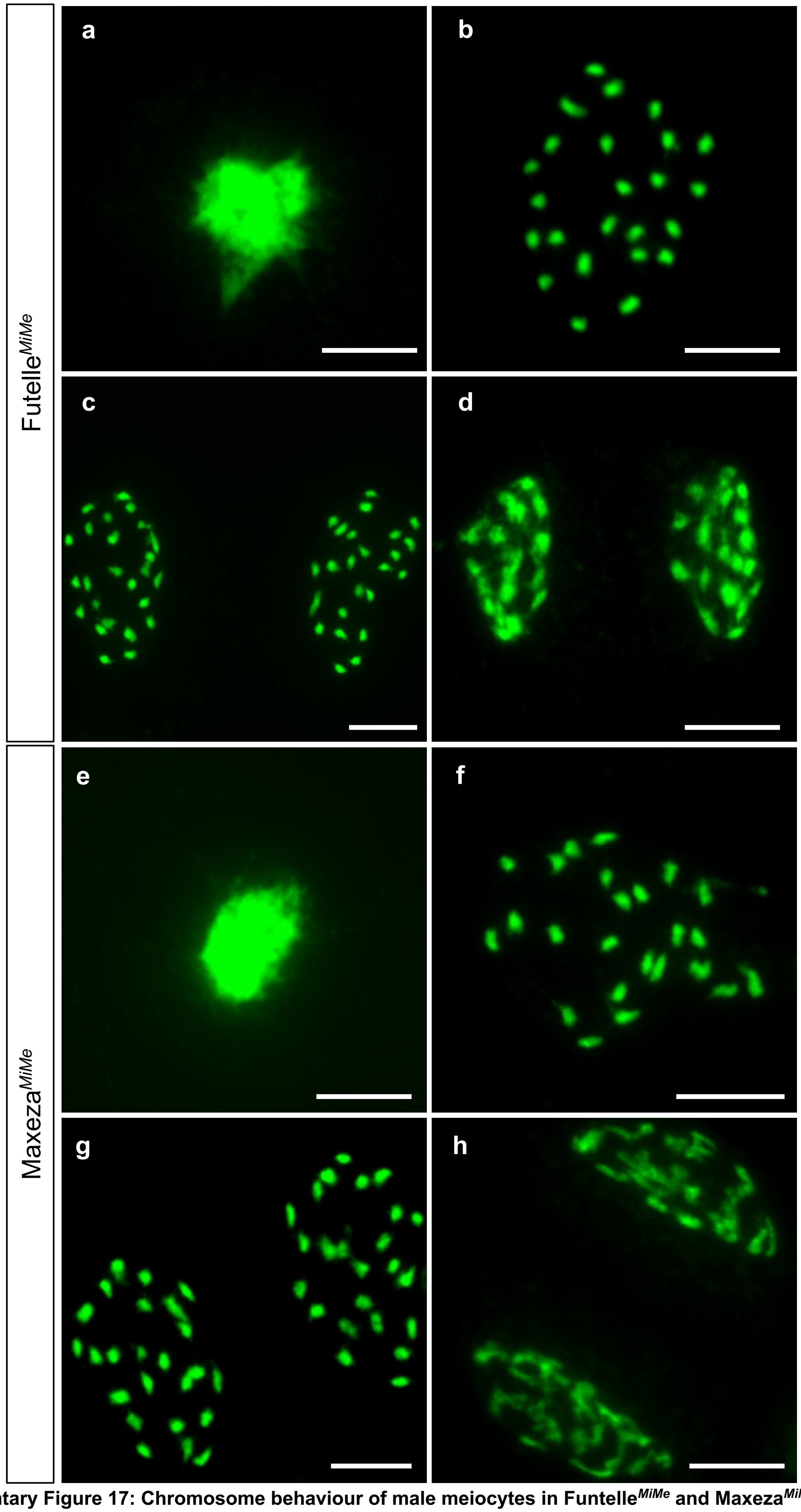

Supplementary Figure 17: Chromosome behaviour of male meiocytes in Funtelle^{MiMe} and Maxeza^{MiMe} mutant.

a-**d**, Chromosome behaviour of *Slspo11-1 rec8 tam* mutant in the Funtelle F1 hybrid background (**a**) Zygotene (n=43); (**b**) Diakinesis (n=25); (**c**) Anaphase I (n=38) and (**d**) Dyad (n=28), Scale bar = 10 µm.

e-**h**, Chromosome behaviour of *Slspo11-1 rec8 tam* mutant in the Maxeza F1 hybrid background (**e**) Zygotene (n=37); (**f**) Diakinesis (n=32); (**g**) Anaphase I (n=26) and (**h**) Dyad (n=35), Scale bar = 10 µm.

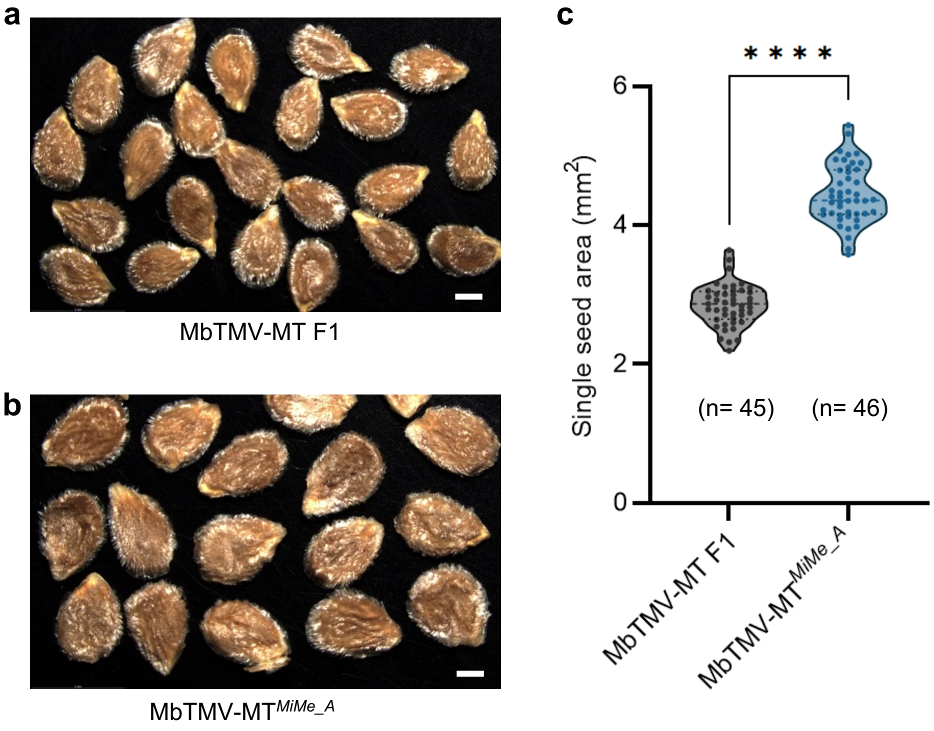

Supplementary Figure 18: Quantitative seed size analysis of seeds collected from MbTMV-MT F1 hybrid and MbTMV-MT^{MiMe_A}.

a, dry mature seeds from MbTMV-MT F1 (n= 45), scale bar = 1mm. **b,** dry mature seeds from MbTMV-MT^{MiMe_A} (n= 46), scale bar = 1mm. **c,** Seed size quantitative analysis in MbTMV-MT F1 hybrid and MbTMV-MT^{MiMe_A}. Seed images were taken using LAS X software and processed using the "threshold" feature of ImageJ (https://imagej.net/software/fiji/downloads). Seed size area was measured using the "Analyze Particles" feature, with a lower limit "1-Infinity mm^2" to exclude any non-seed material. The final data was analyzed using Microsoft Excel and GraphPad Prism 9 software. MbTMV-MT F1 (n=45) and hybrid MbTMV-MT^{MiMe_A} (n=46). The statistical analysis of single seed area was conducted with unpaired t-test (Two-tailed) and **** means $P < 0.0001$. The exact P value = 1.24×10^{-34}.

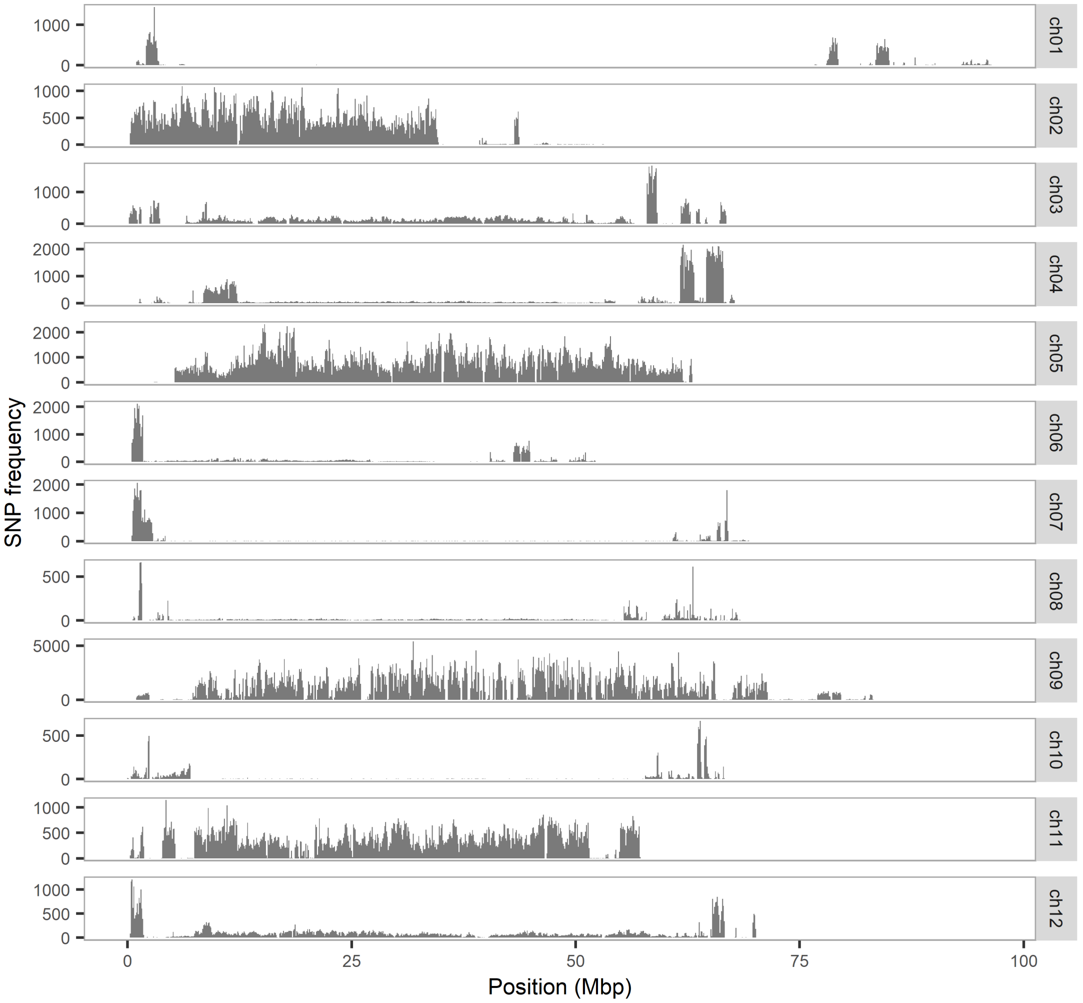

Supplementary Figure 19: Distribution of segregating markers between Moneyberg-TMV (MbTMV) and Micro-Tom (MT).

Only homozygous MT SNPs relative to the MbTMV reference were selected as markers. These markers should not overlap with any SNPs in MbTMV raw reads that were mapped against the MbTMV reference genome. Low SNP regions can be explained due identical sequence by descent (Moneymaker is part of the recent pedigree of both Moneyberg-TMV and Micro-Tom) and, for example the pericentromere of chromosome 7, due to previously reported selective sweeps in domesticated tomato. High SNP density such as in chromosome 9 is due to the introgression of TMV resistance into MbTMV from *S. peruvianum*.

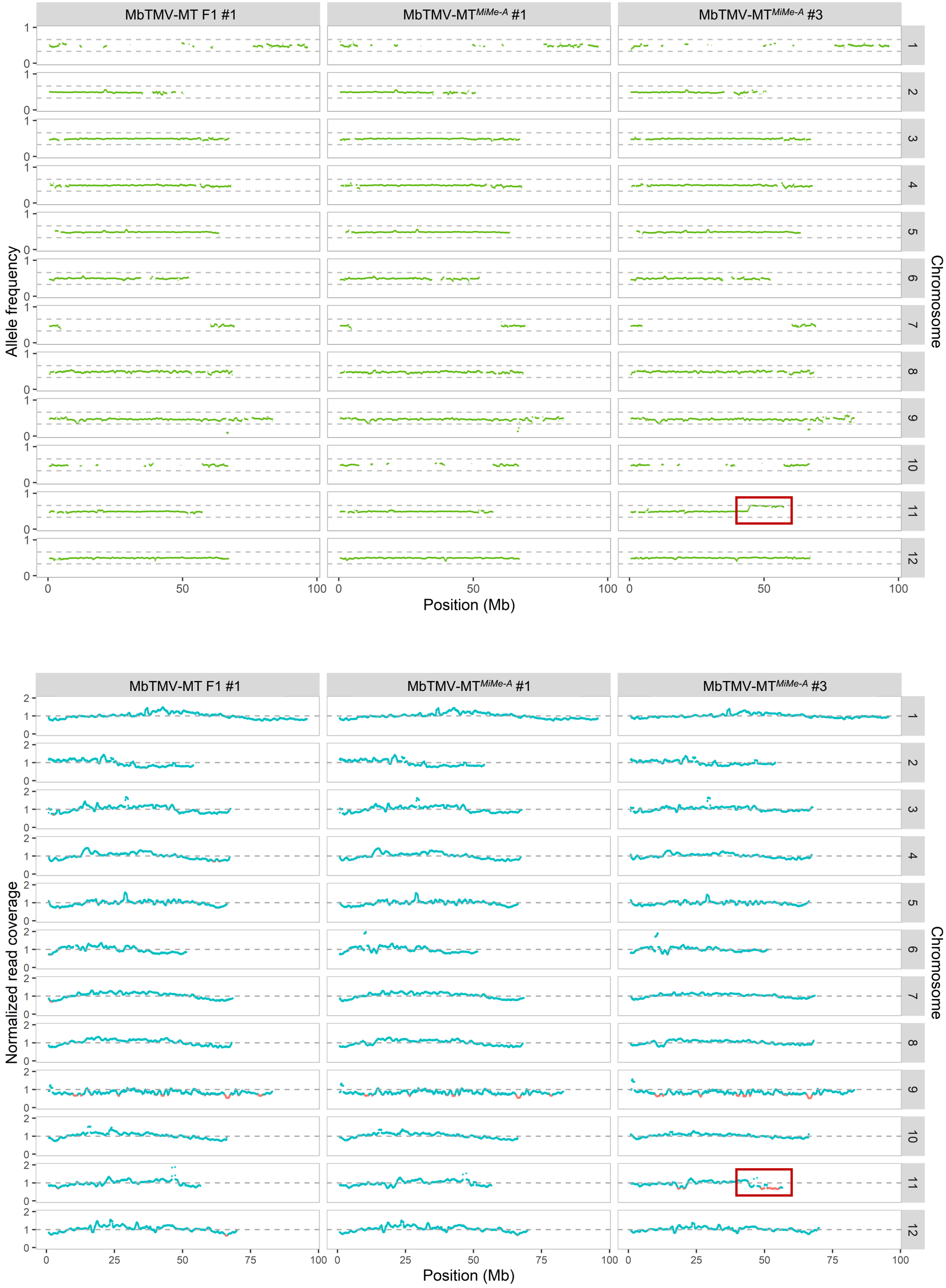

Supplementary Figure 20: Allele frequency and read coverage distribution show chromosome fragmentation in one *MiMe* offspring.

Allele frequency of 0 and 1 indicate fully MbTMV or MT genotype, respectively. The first two columns show samples without truncation (MbTMV-MT F1#1 and MbTMV-MT^{MiMe-A} #1) and the third column for a sample with truncation (MbTMV-MT^{MiMe-A} #3). Top: A truncation was observed as a chromosomal segment (red box on chromosome 11) with allele frequency diverged from 0.5 to 0.66. Unlike in F2 plants where a change of frequency from 0.5 to either 0 or 1 would suggest meiotic recombination, the frequency of 0.66 in a tetraploid plant suggests one of the MbTMV derived chromosome 11 copies has been truncated. Bottom: the divergence from 0.5 allele frequency was corroborated by reduced genome coverage (red dots) in the same region of chromosome 11 compared with the genome average (blue dots). Both conditions (diverged allele frequency and reduced coverage) should be satisfied to differentiate it from genetic deletions in the Micro-Tom genome relative to the MbTMV reference.

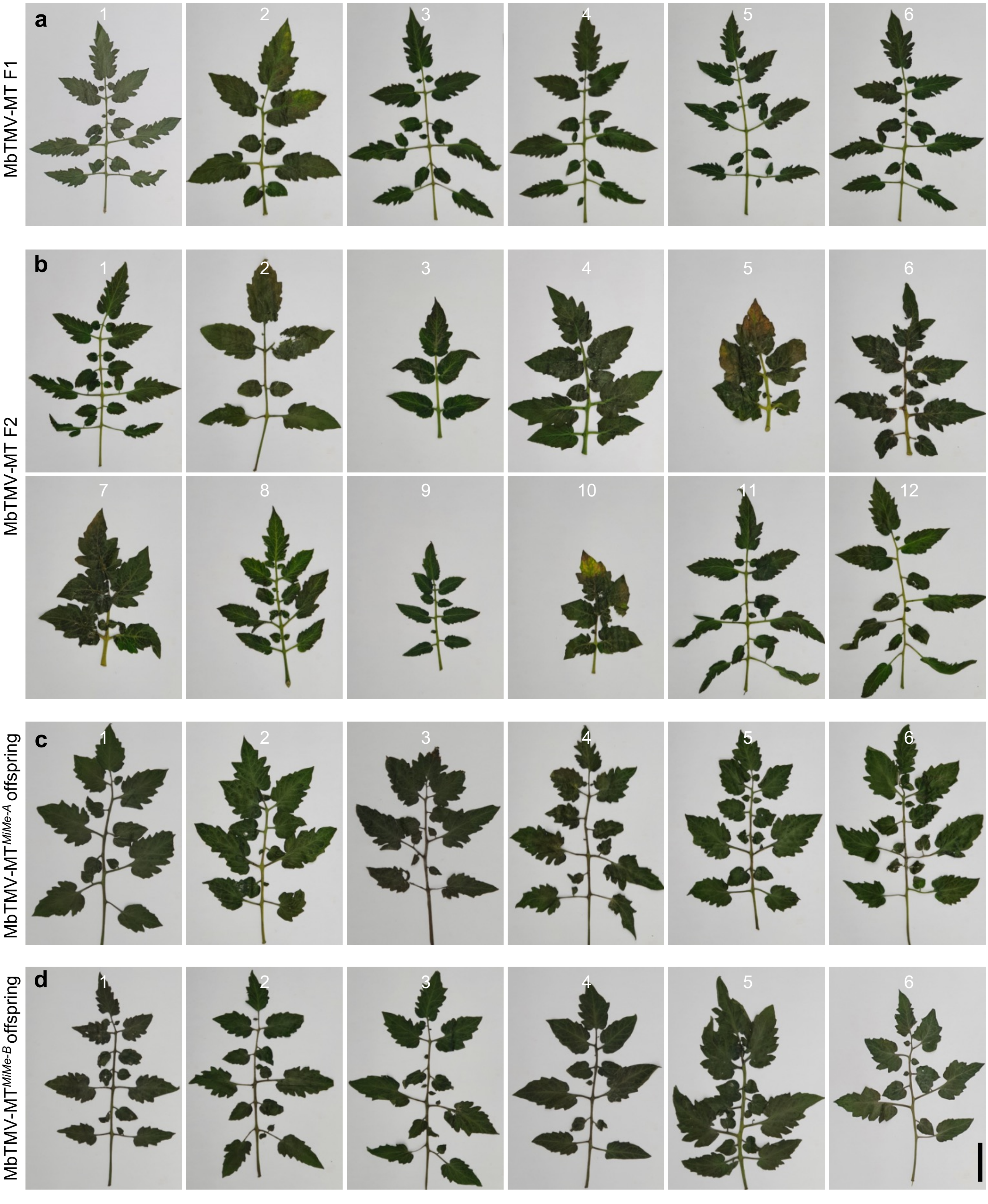

Supplementary Figure 21: The leaf morphology of MbTMV-MT F1 plants, MbTMV-MT F2 plants, MbTMV-MT^{MiMe-A} offspring plants and MbTMV-MT^{MiMe-B} offspring plants.

Comparison of leaf phenotypes in individual plants from MbTMV-MT F1 (**a**, 6 plants), MbTMV-MT F2 (**b**, 12 plants), MbTMV-MT^{MiMe-A} offspring (**c**, 6 plants) and MbTMV-MT^{MiMe-B} offspring (**d**, 6 plants). Scale bars= 6 cm.

a b c

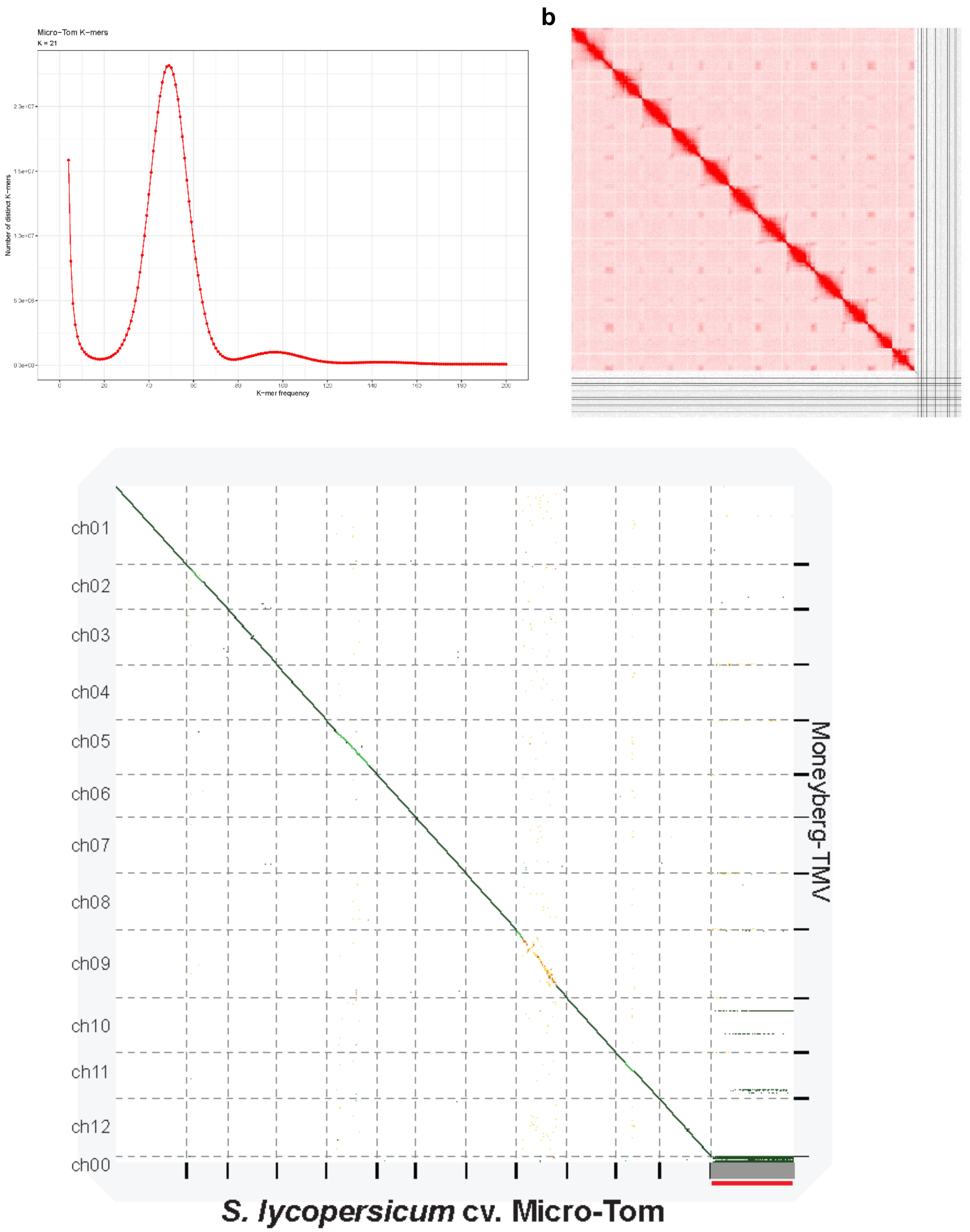

Supplementary Figure 22. Chromosome scale *de novo* genome assembly of Micro-Tom.
a, K-mer Analysis Toolkit (KAT) results of the K-mer distribution in Micro-Tom HiFi data. *S. lycopersicum* cv. Micro-Tom HiFi K-mer frequency distribution where K = 21. **b**, Hi-C contact plot of Micro-Tom hifiasm assembly scaffolded with Omni-C data. Hi-C contact plots are created by Salsa2 and Juicebox. Vertical and horizontal lines represent scaffold borders. **c**, *S. lycopersicum* cv. Micro-Tom genome aligned against *S. lycopersicum* cv. Moneyberg-TMV (van Rengs et al., 2022). Dotplots are generated using D-genies (Cabanettes and Klop, 2018). Horizontal grey dashed lines represent MbTMV chromosome borders. Vertical grey dashed lines represent Micro-Tom chromosome borders. Unplaced contigs aligned to Moneyberg-TMV “ch00” are marked by a red line.

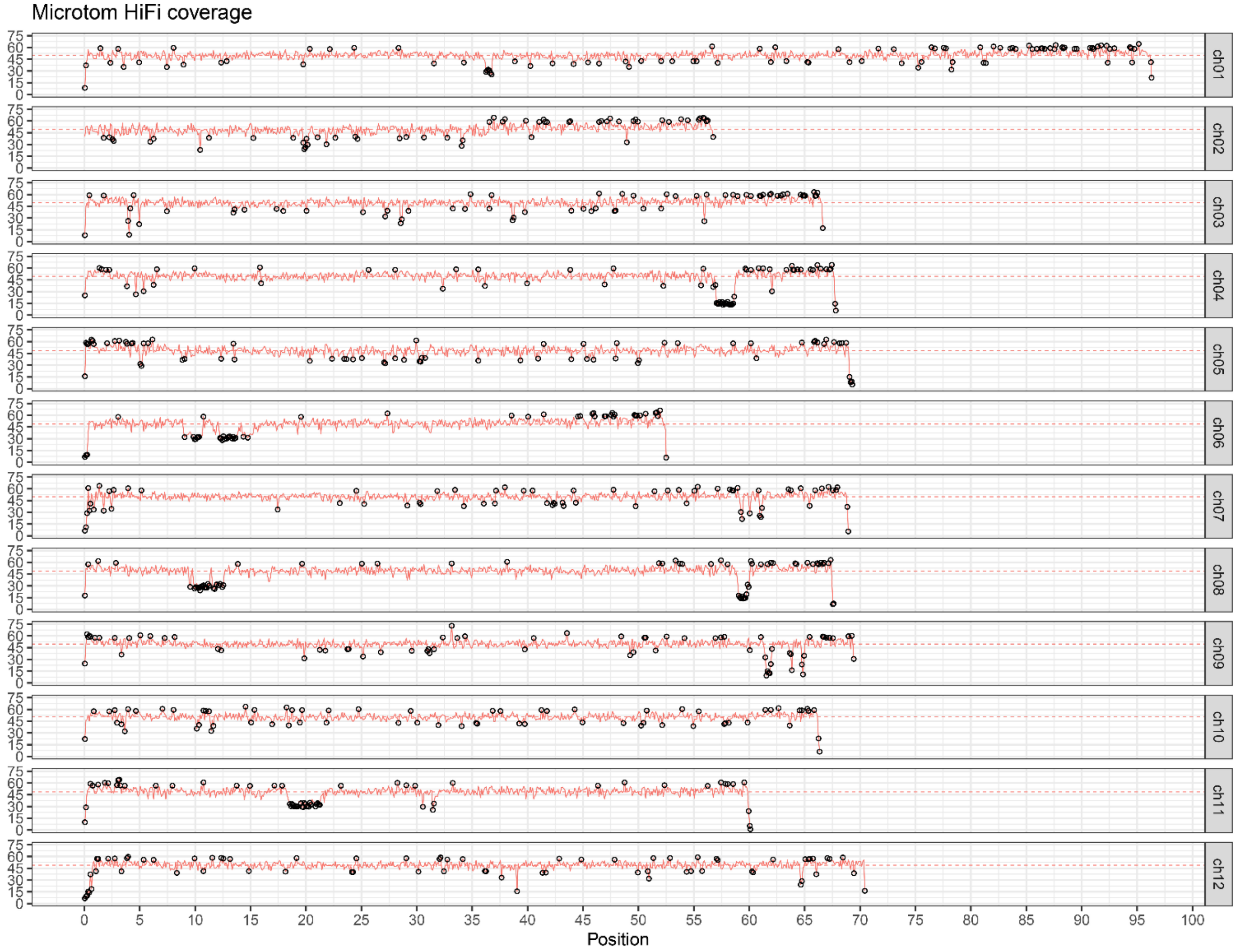

Supplementary Figure 23. Characterization and validation of the 12 Micro-Tom chromosomes.
Characterization and validation by read coverage analysis in 100kb windows plotted over the genomic position on the genome (Red solid lines represents HiFi read coverage). Dashed horizontal line represents mean coverage. Black circles represent coverage outliers (<2.5% and >97.5% percentiles). X-axis is chromosome position in Mbp.

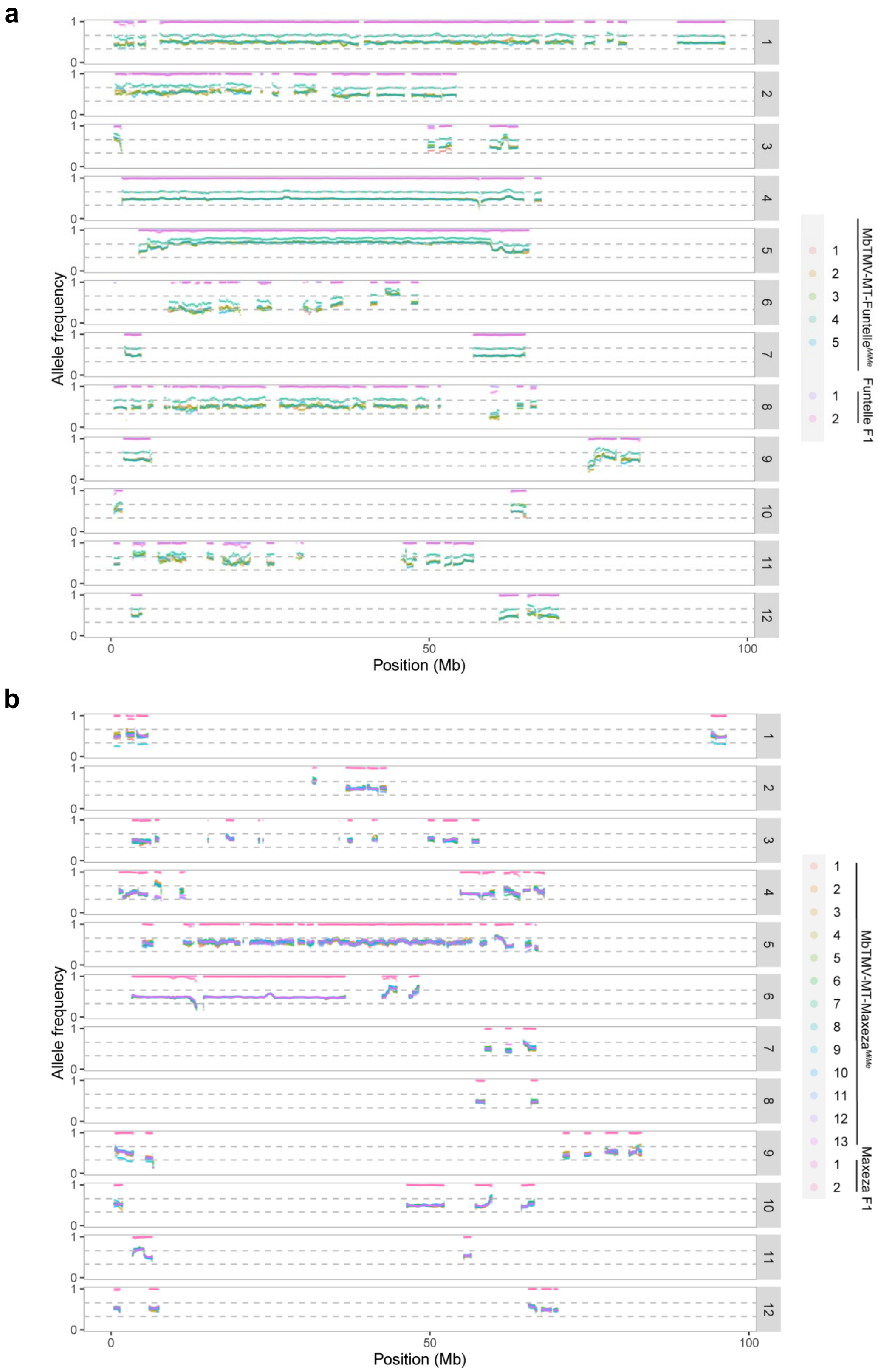

Supplementary Figure 24: Allele frequency genotyping in (a) MbTMV-MT-Funtelle^{MiMe} 4-Hap plants and (b) MbTMV-MT-Maxeza^{MiMe} 4-Hap plants.

Allele frequencies indicate fully MbTMV-MT (allele frequency = 0) or fully Funtelle/Maxeza (allele frequency = 1) genotypes. In (a) SNPs that are homozygous and private to Funtelle, and are polymorphic to both MbTMV and MicroTom were used to compute allele frequency; in (b) SNPs that are homozygous and private to Maxeza, and are polymorphic to both MbTMV and MicroTom were used to compute allele frequency. Allele frequencies of 0.5 along the chromosomes are expected in 4-Hap plants due to equal parental contributions to the tetraploid plants. Gaps in the lines are regions with no polymorphism between MbTMV-MT and the respective commercial hybrid. Allele frequency of 0.5 indicates equal genomic contributions in the tetraploid plants. The only two samples in (a) with allele frequency of 1 are Funtelle F1 hybrid controls, while the only two samples in (b) with allele frequency of 1 are Maxeza F1 hybrid controls.

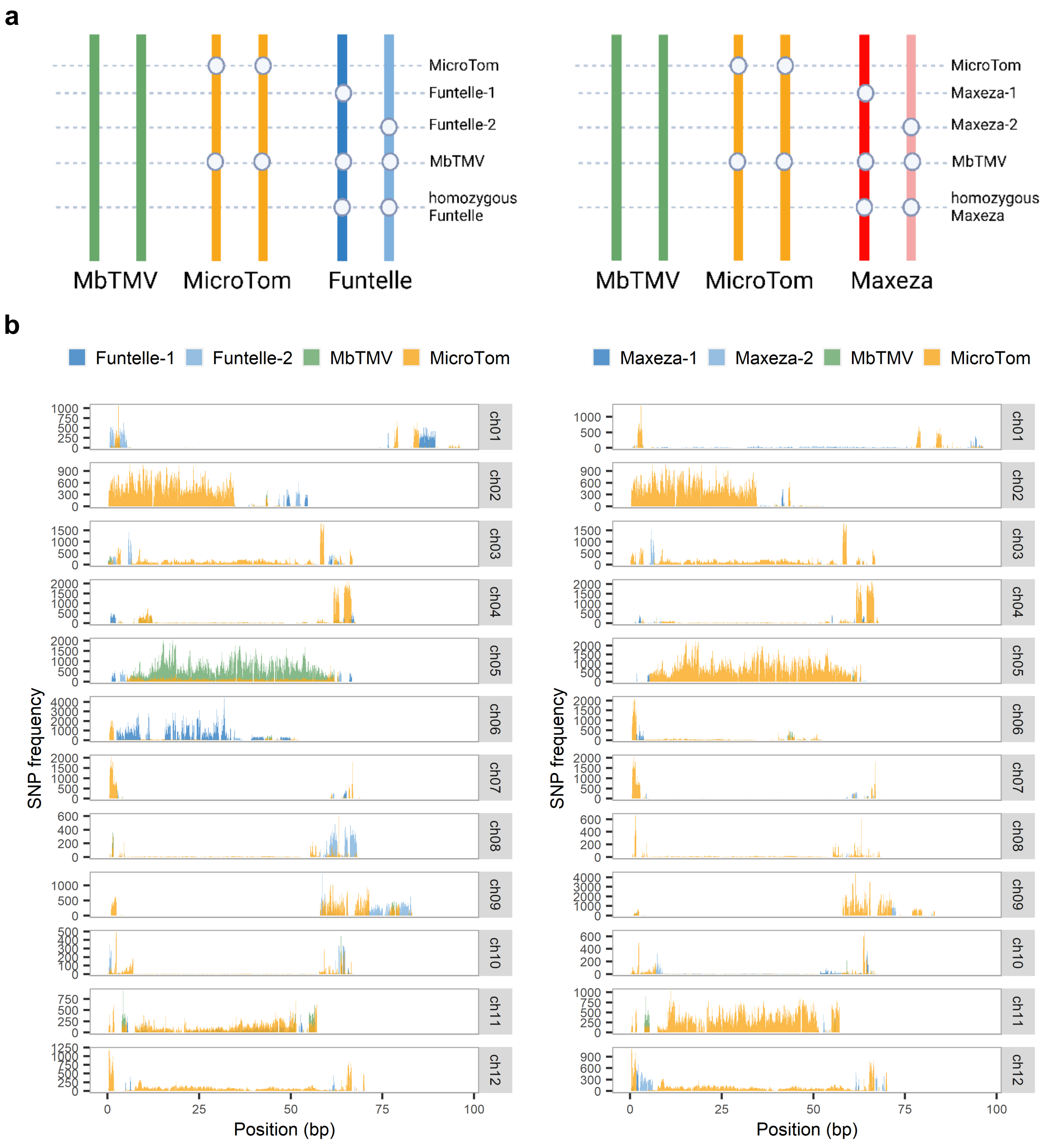

Supplementary Figure 25: Unique markers in tetraploid *MiMe* haplotypes.

a, Diagram on the selection of unique markers per haplotype (Left: Mb-TMV, Micro-Tom, Funtelle haplotype-1 and Funtelle haplotype-2; Right: Mb-TMV, Micro-Tom, Maxeza haplotype-1 and Maxeza haplotype-2)

b, Distribution of unique markers between four different haplotypes (Left: Mb-TMV, Micro-Tom, Funtelle haplotype-1 and Funtelle haplotype-2; Right: Mb-TMV, Micro-Tom, Maxeza haplotype-1 and Maxeza haplotype-2)

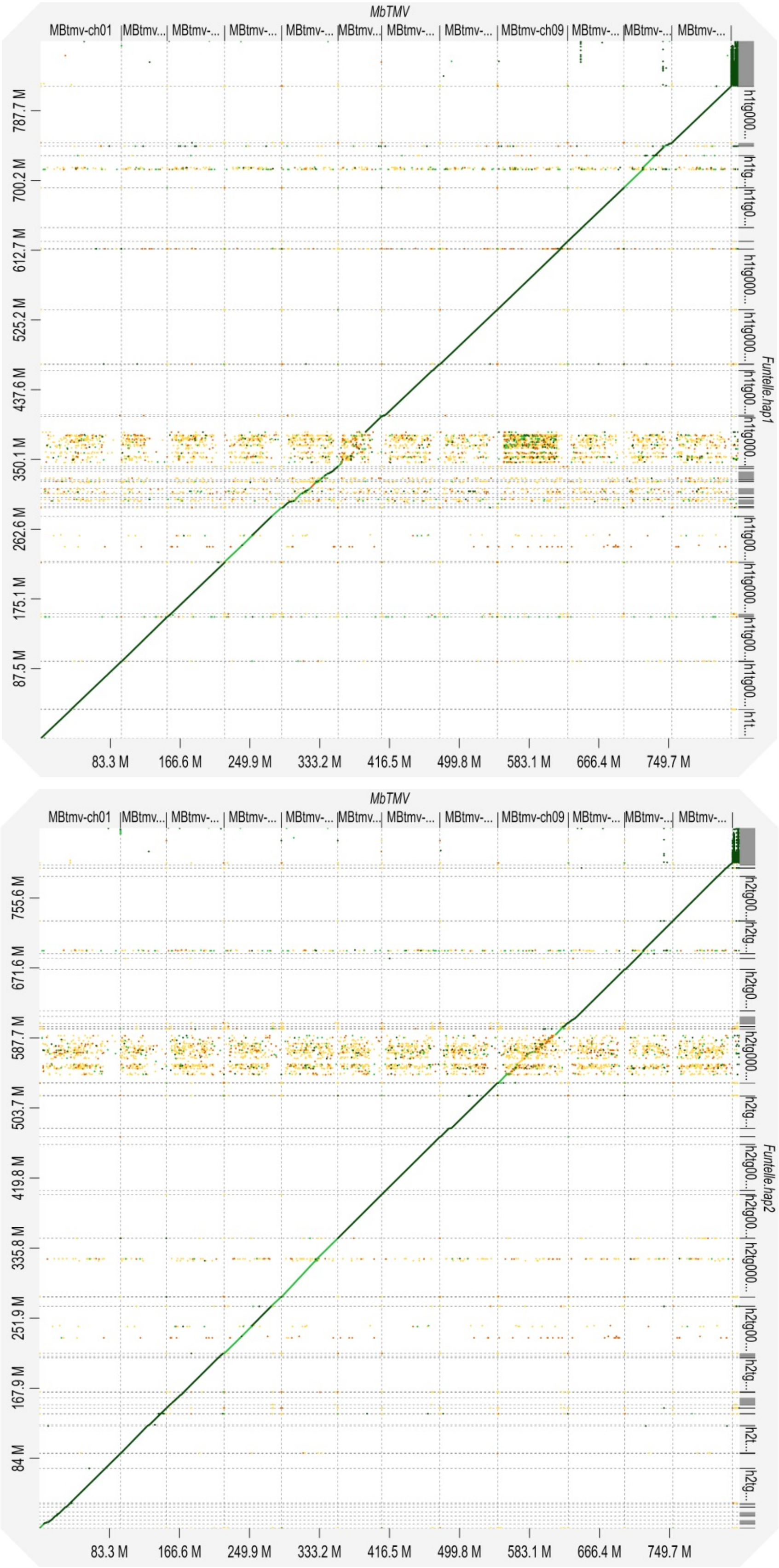

Supplementary Figure 26: Alignment of Funtelle haplotype-specific contigs against the MbTMV assembly.
Raw contigs from a *hifiasm* assembly of Funtelle HiFi reads were aligned against the Mb-TMV reference genome sequence (van Rengs et al., 2022) and visualized using D-Genies. Most contigs aligned well or are syntenic with the MbTMV assembly except chromosome 6 of Funtelle-1, which has an *Mi-1* introgression not present in Mb-TMV, and chromosome 9 of Funtelle-2, which does not have the TMV introgression.

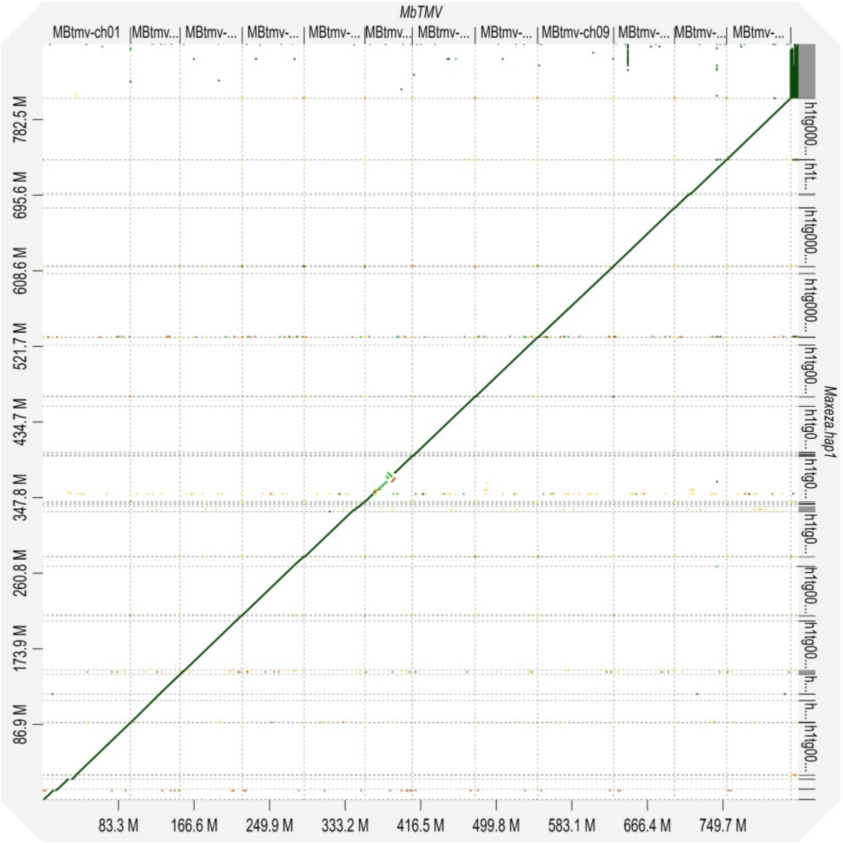

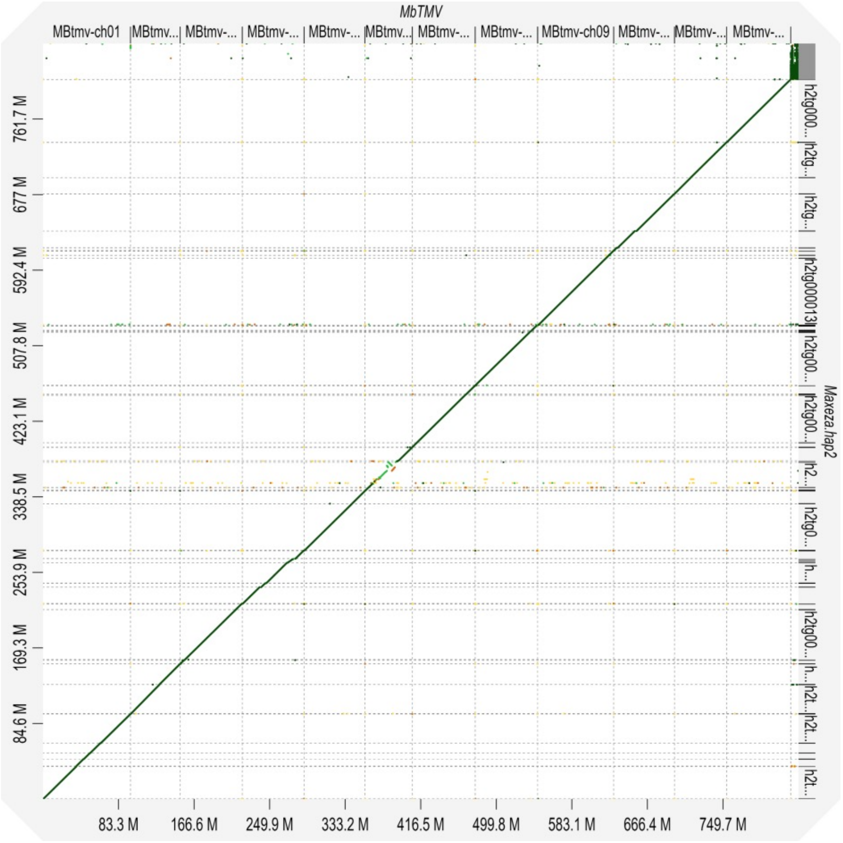

Supplementary Figure 27: Alignment of Maxeza haplotype-specific contigs against the MbTMV assembly.
Raw contigs from a *hifiasm* assembly of Maxeza HiFi reads were aligned against the Mb-TMV reference genome sequence (van Rengs et al., 2022). Most contigs aligned well and are syntenic with the MbTMV assembly.

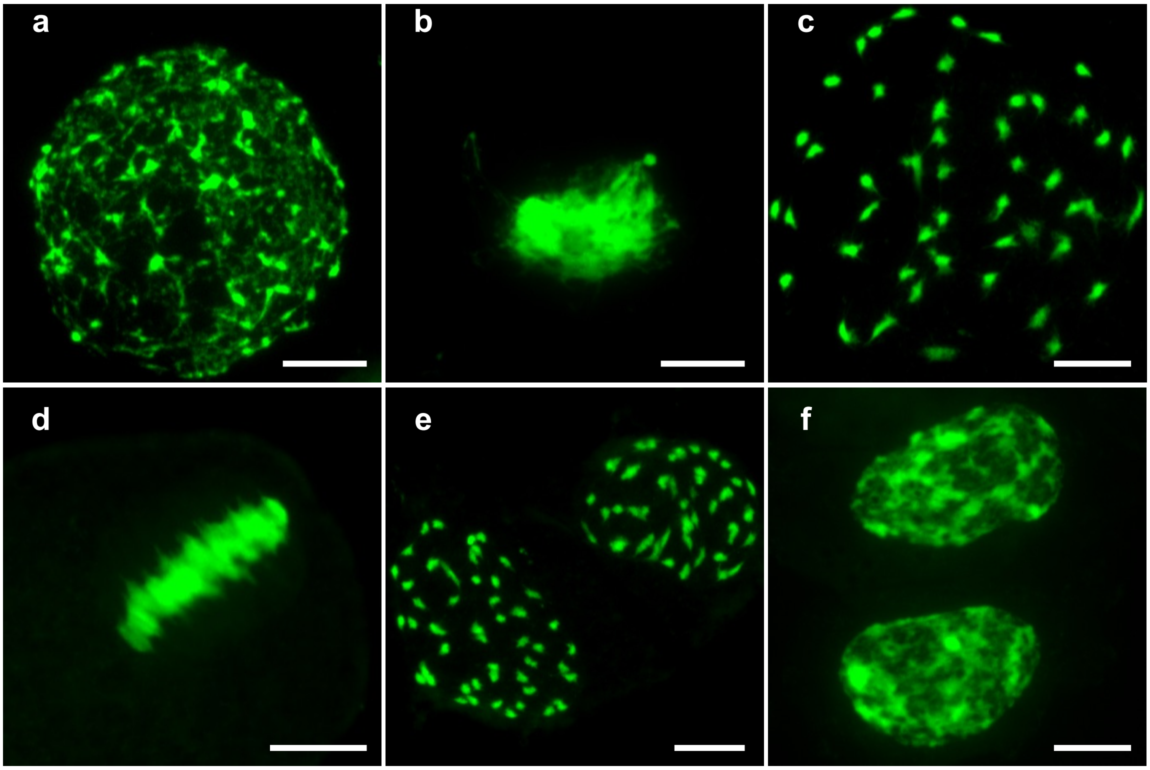

Supplementary Figure 28: Chromosome behaviour of male meiocytes in the MbTMV-MT-Maxeza$^{MiMe\text{-}6}$ plant.
Chromosome behaviour of male meiocytes in the MbTMV-MT-Maxeza$^{MiMe\text{-}6}$ mutant plant during (**a**) Leptotene (n=43); (**b**) Zygotene (n=48); (**c**) Diakinesis (n=31); (**d**) Metaphase I (n=22); (**e**) Anaphase I (n=42) and (**f**) Dyad (n=51). Scale bar = 10 µm.

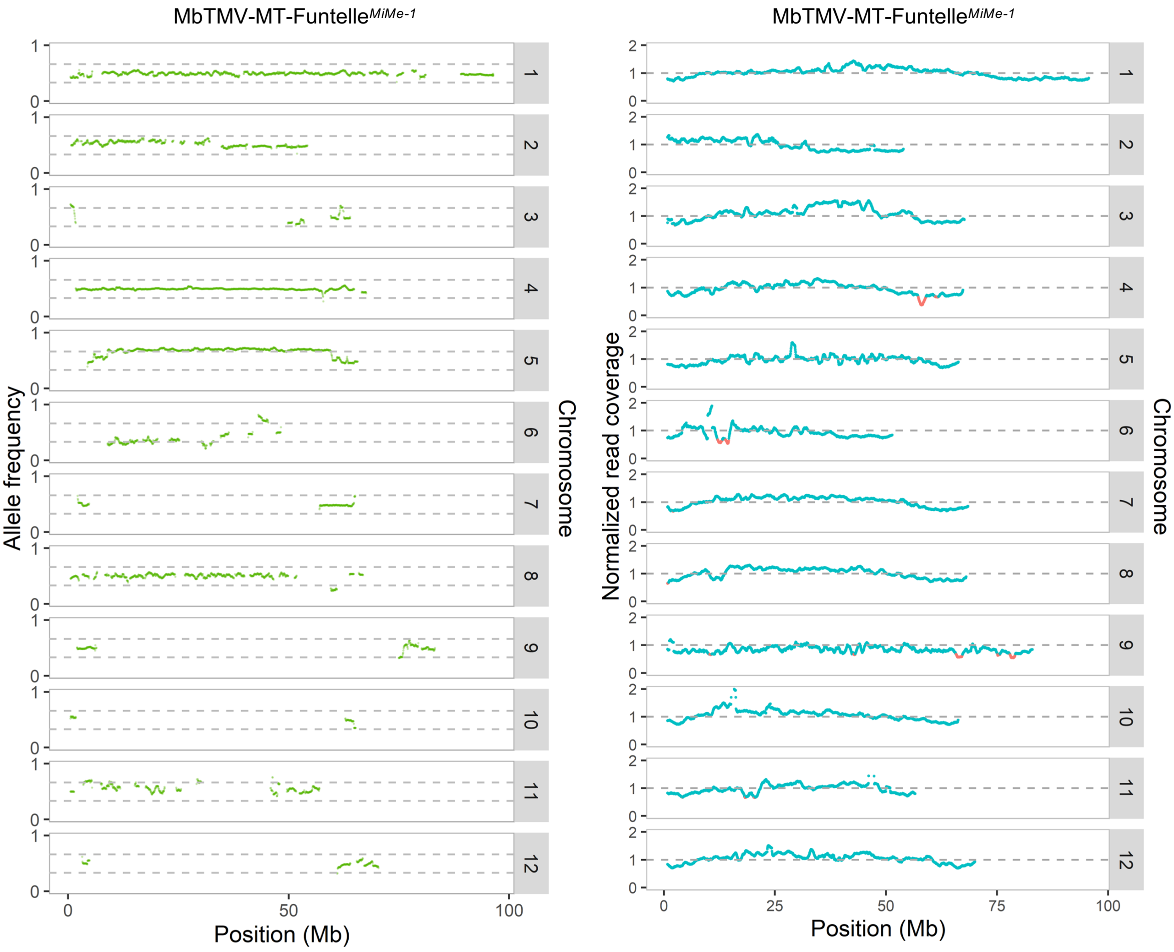

Supplementary Figure 29: Allele frequency and read coverage distribution show normal chromosome behaviour in MbTMV-MT-Funtelle^{MiMe-1}.

Allele frequency of 0 indicates fully MbTMV-MT genotype and allele frequency of 1 indicates fully Funtelle genotype. As shown here, an allele frequency of 0.5 along the chromosomes is expected in a normal 4-Hap plant due to equal parental contributions to the tetraploid plant. This normal chromosome behavior is further confirmed by the normalized read coverage plot which suggests no evidence for chromosome truncations.

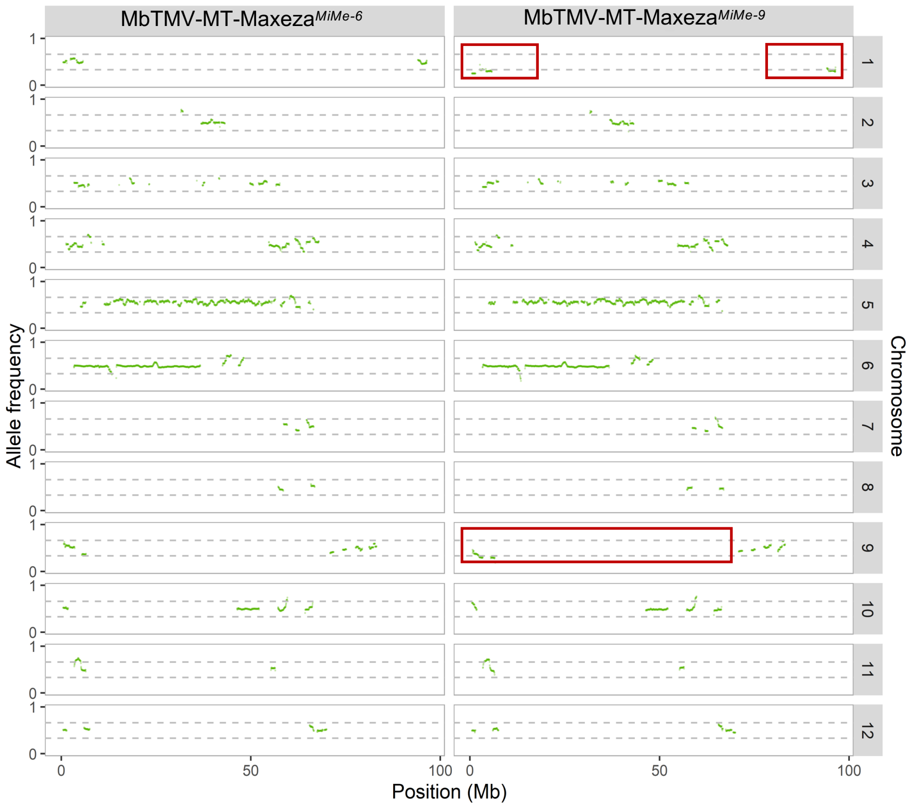

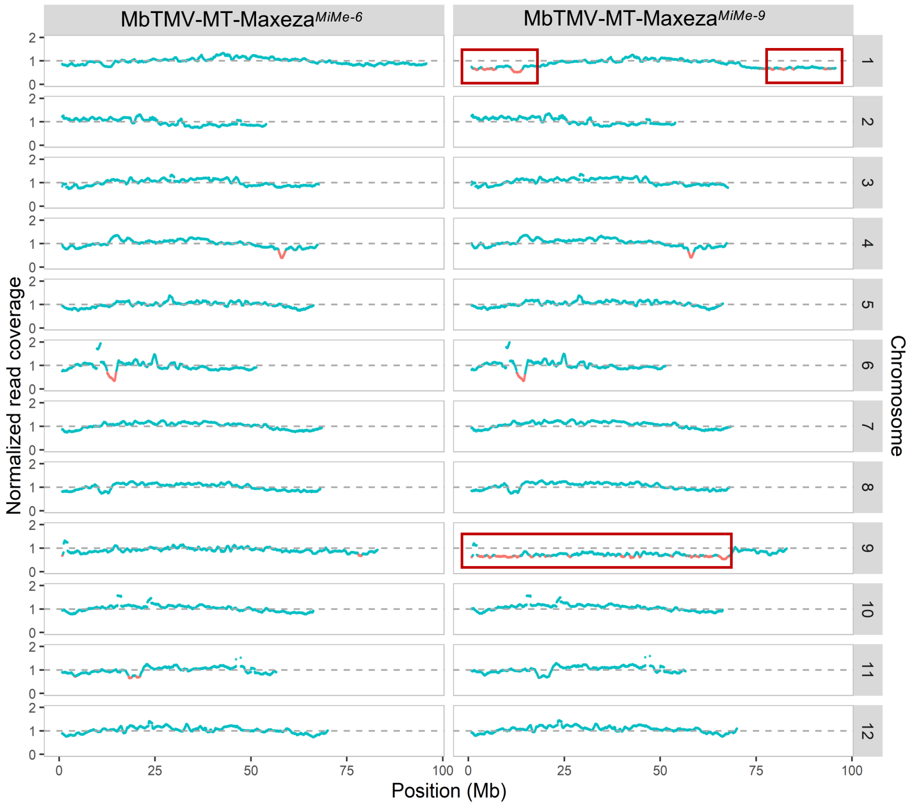

Supplementary Figure 30: Allele frequency and read coverage distribution show normal chromosome behaviour in MbTMV-MT-Maxeza$^{MiMe\text{-}6}$ and abnormal chromosome behavior in MbTMV-MT-Maxeza$^{MiMe\text{-}9}$.

Allele frequency of 0 indicates fully MbTMV-MT genotype and allele frequency of 1 indicates fully Maxeza genotype. An allele frequency of 0.5 along the chromosomes is expected in a normal 4-Hap plant due to equal parental contributions to the tetraploid plant (see MbTMV-MT-Maxeza$^{MiMe\text{-}6}$). In contrast single chromosome truncation could be an explanation for allele frequency divergences to 0.33 or 0.66. In the case of MbTMV-MT-Maxeza$^{MiMe\text{-}9}$ we observed divergence on chromosome 1 and 9 to allele frequency of 0.33 (red boxes, top right) indicating potential missing genomic segments from the MbTMV-MT parent. This was further confirmed (red boxes, bottom right) by the reduced genome coverage (red dots) compared with the genome average (blue dots). Both divergence in allele frequency and normalized read coverage are required to differentiate it from deletions in any of the Maxeza haplotypes relative to the MbTMV reference.

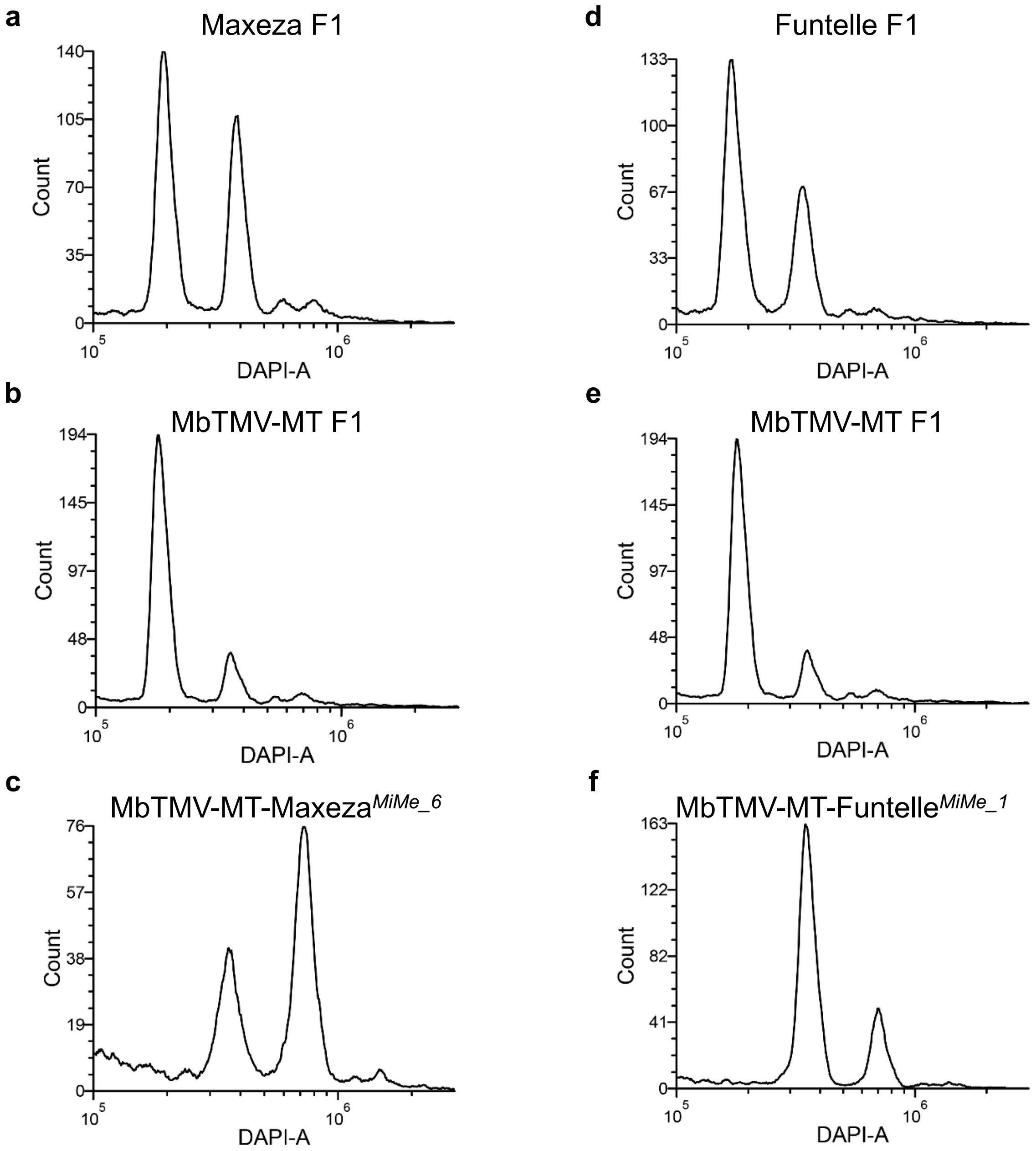

Supplementary Figure 31: The ploidy level of wild type diploid and 4-haplotype tetraploid plants.
Flow cytometry based ploidy level detection of (**a**) diploid Maxeza F1, (**b**) diploid MbTMV-MT F1, (**c**) tetraploid MbTMV-MT-Maxeza^{MiMe_6}, (**d**) diploid Funtelle F1, (**e**) diploid MbTMV-MT F1 and (**f**) tetraploid MbTMV-MT-Funtelle^{MiMe_1}. The same MbTMV-MT F1 is used twice in this panel to facilitate vertical comparison.

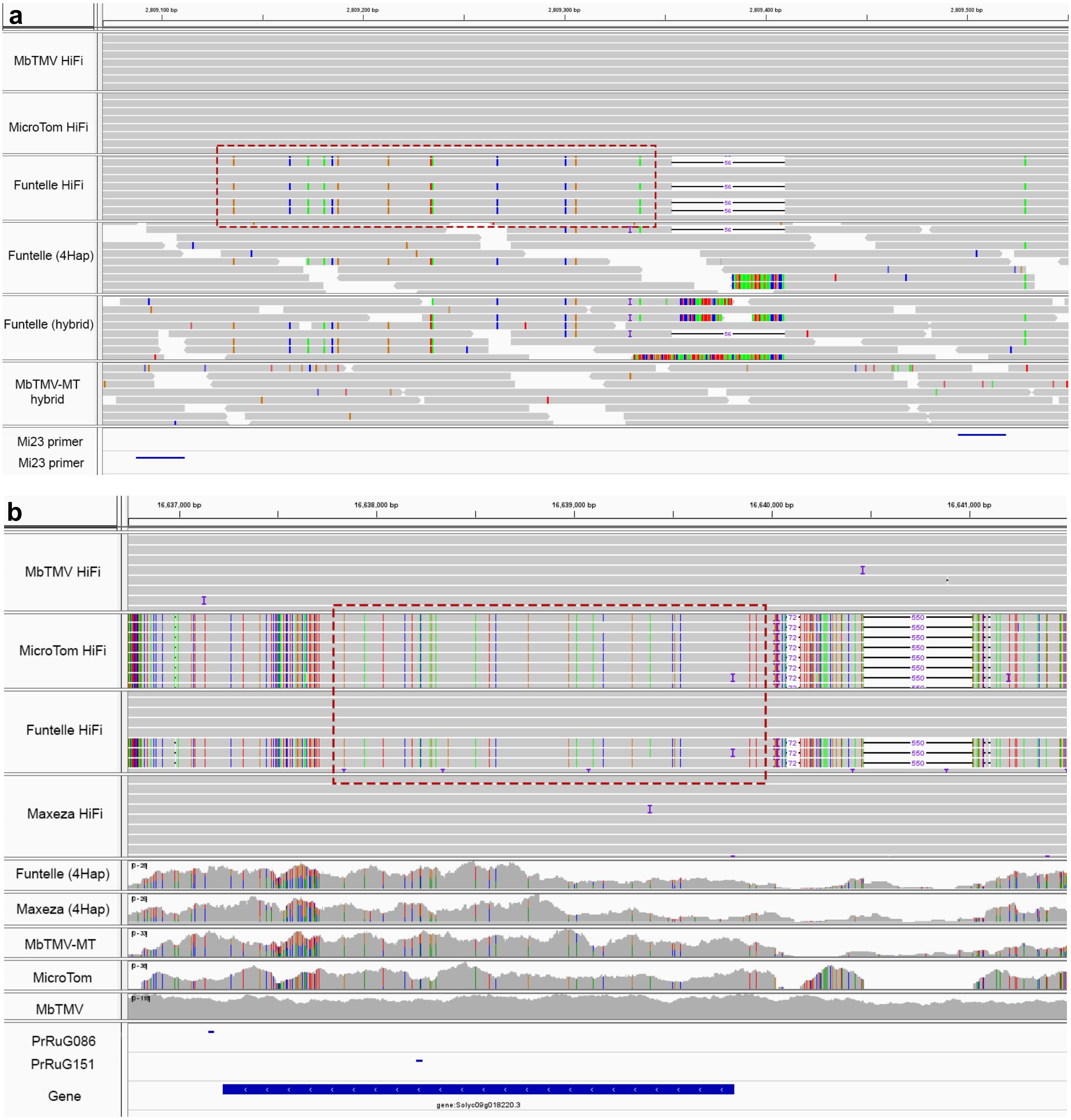

c

	MbTMV	Micro-Tom	Funtelle-1	Funtelle-2	Maxeza-1	Maxeza-2
Tm-2²	99.99	31.88	99.99	31.88	99.99	99.99
Tm-2	97.7	99.96	97.7	99.86	97.7	97.7

Supplementary Figure 32: IGV visualization of *Mi-1* and *Tm-2²* introgression in control and 4-Hap plants.
a, The Integrative Genomics Viewer (IGV) visualization of *Mi-1*-linked 56-bp deletion (Garcia et al., 2007; Devran et al., 2016). Funtelle contains heterozygous introgression of *Mi-1* indicated by the haplotype spanning both the deletion and the SNPs upstream the deletion. **b**, IGV visualization of *Tm-2²*, plotted with the primers discriminating resistant and susceptible alleles (Lanfermeijer et al., 2005). Micro-Tom has no *Tm-2²* introgression while Funtelle is heterozygous for the *Tm-2²* introgression while both MbTMV and Maxeza are homozygous for the introgression. SNPs within the red box in **a** and **b** were used to genotype the 4-Hap samples as shown in Figure 2e. **c**, Percentage of matched bases (relative to gene size) between the parental assembly and the *Tm-2²* and *Tm-2* (susceptible) sequences.

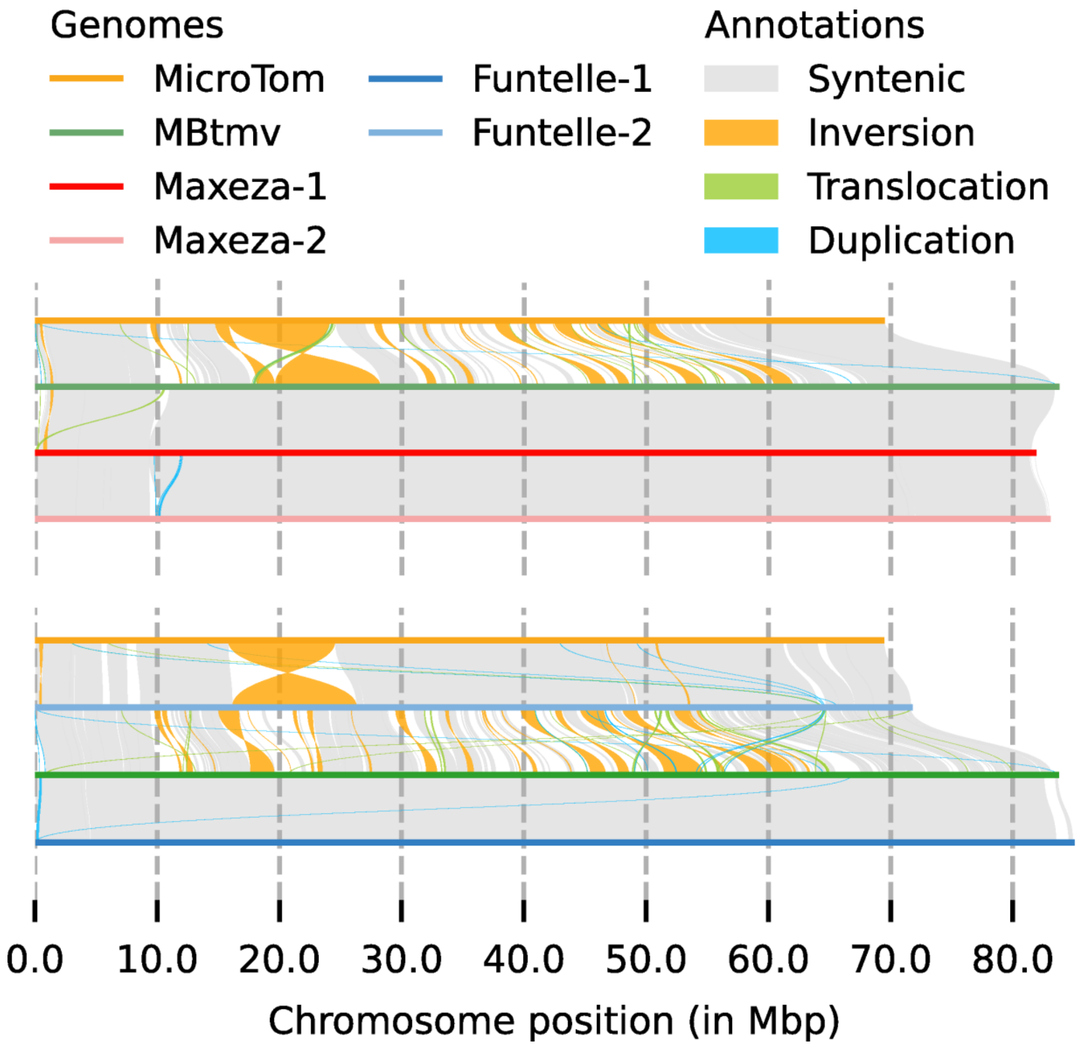

Supplementary Figure 33: Synteny plot showing the *Tm-2*^{2} introgression in chromosome 9 of the parental genomes. The *Tm-2*^{2} gene is located within a 64.1 Mbp introgression from *Solanum peruvianum* that provides tomato plants with resistance to TMV (van Rengs et al., 2022). Here whole chromosome alignment and SyRI based structural comparison of chromosome 9 sequences compares MbTMV (homozygous for the introgression), Micro-Tom (no introgression), Maxeza (both Maxeza-1 and Maxeza-2 haplotypes contain the introgression) and Funtelle (only Funtelle-1 haplotype contains the introgression).

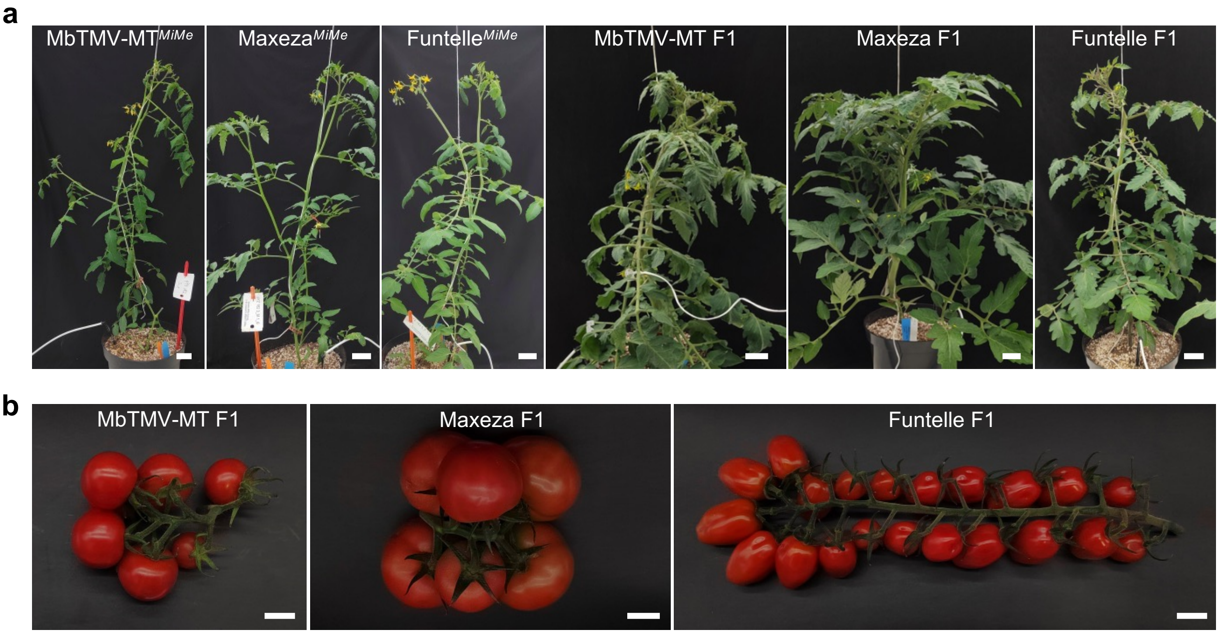

Supplementary Figure 34: Plant morphology of hybrid *MiMe* plants and F1 hybrid plants.

a, Young plant morphology of hybrid *MiMe* plants and control F1 hybrid plants (From left to right: MbTMV-MT^{MiMe}, Maxeza^{MiMe}, Funtelle^{MiMe}, MbTMV-MT F1, Maxeza F1 and Funtelle F1). Scale bar = 5cm.

b, Mature fruits collected from the three F1 hybrid genotypes (MbTMV-MT F1, Maxeza F1 and Funtelle F1). Scale bar = 2cm.

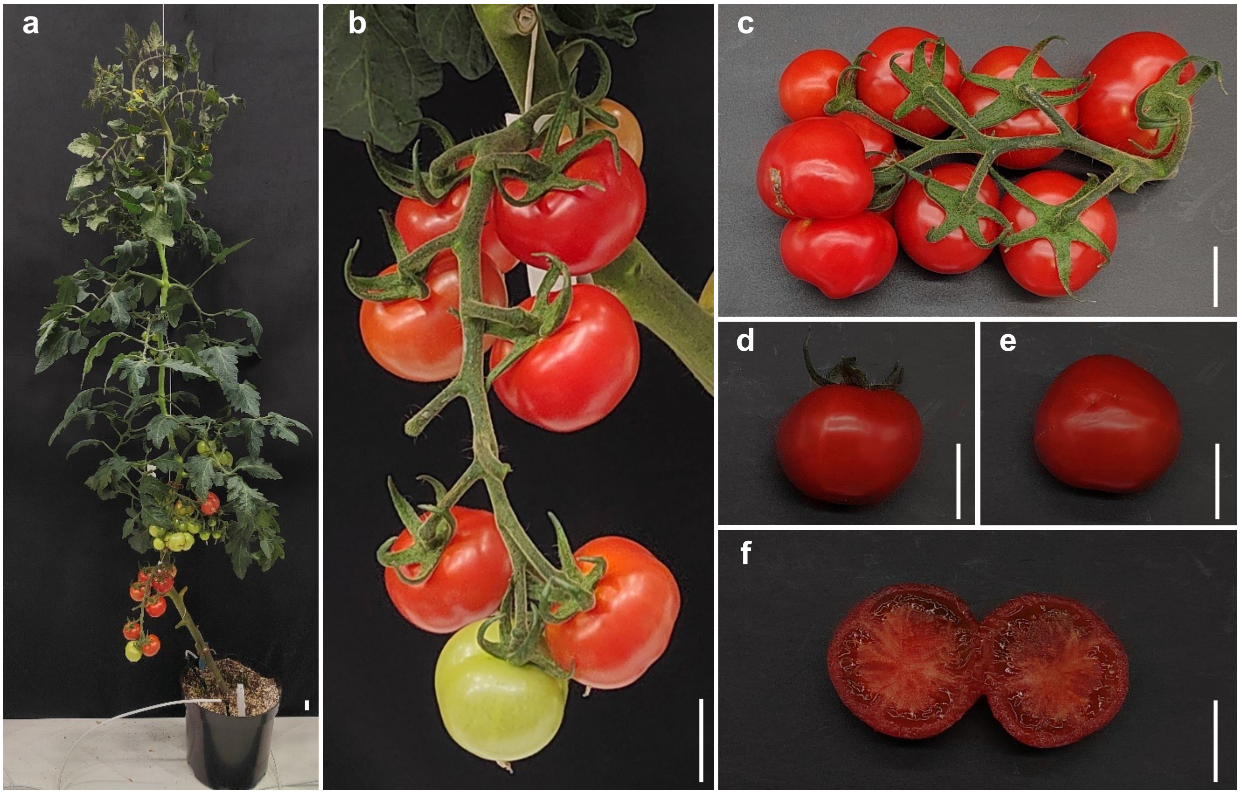

Supplementary Figure 35: Plant morphology of the MbTMV-MT-Maxeza*MiMe-6* mutant.
a, Whole plant morphology of MbTMV-MT-Maxeza*MiMe-6*. Scale bar = 2.5 cm.
b, The structure of a branch with ripening tomato fruits. Scale bar = 2.5 cm.
c, A single branch with fully matured fruits. Scale bar = 2.5 cm.
d, A fully ripened fruit with pedicel and sepal. Scale bar = 2.5 cm.
e, A fully ripened fruit without pedicel and sepal. Scale bar = 2.5 cm.
f, Transverse anatomical view of single fruit. Scale bar = 2.5 cm.

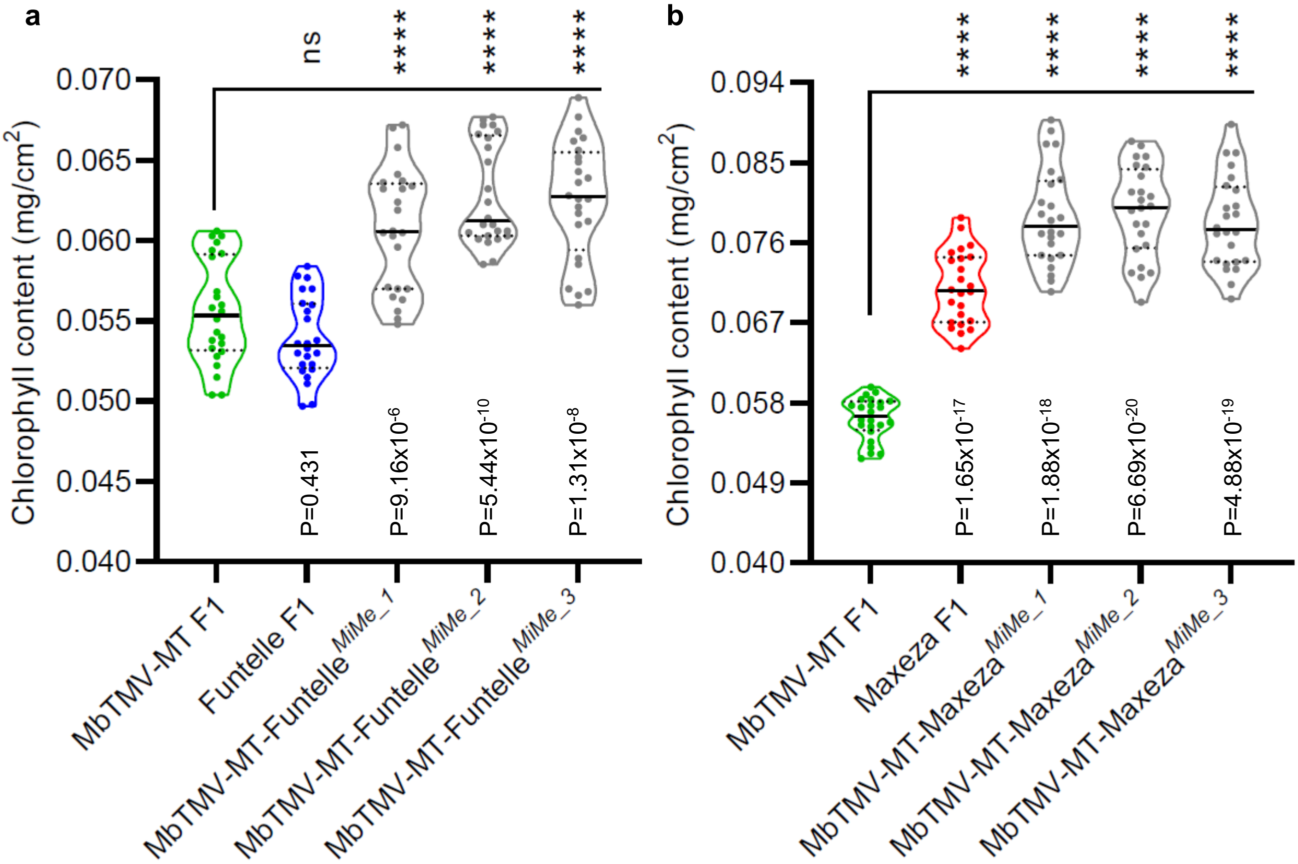

Supplementary Figure 36: Leaf chlorophyll content measurement in control and 4-Hap plants.

a, Leaf chlorophyll content measurements in control hybrid and 4-Hap (MbTMV-MT-Funtelle^{MiMe}) plants. **b,** Leaf chlorophyll content measurements in control hybrid and 4-Hap (MbTMV-MT-Maxeza^{MiMe}) plants. For each plant, leaves were counted from the shoot apical meristem towards the ground to identify the fifth leaf (which was in all cases a mature leaf) which was used for analysis. Four different leaflets on each leaf were measured for chlorophyll content and six measurements were performed per single leaflet (totaling 24 measurements per genotype). The statistical analysis of leaf chlorophyll content was conducted with the ordinary one-way ANOVA followed by Tukey's multiple comparisons test. Definition of statistical significance: $P < 0.05$. 'ns' means no significance, and **** means < 0.0001.

Supplementary Table 1. Plasmids used for agrobacterium transformation in this study.						
**Plasmid name**	**Targted genes**	**gene ID**	**Chromosome number**	**Guide RNA number**	**Guide RNA sequence**	**Genetic background**
pDIRECT_22C	-	-	-	-	-	-
pYZ14	*TAM*	*Solyc11g005090*	11	2	AACAGTAATGCAGTTTCCTCAGG, CGACACAGTGAGAAGTCCGGAGG	Micro-Tom
pYZ178_1	*OSD1*	*Solyc10g080400*	10	2	AGGTGGCATTGAGATCTTCGAGG, TACAGACTACAGCAGAAGCAGGG	Micro-Tom
pYZ178_2				1	TACAGACTACAGCAGAAGCAGGG	Micro-Tom
pYZ1	*TAM, REC8, SPO11-1*	*Solyc11g005090, Solyc06g074860-Solyc06g074870-Solyc06g074880, Solyc07g065430-Solyc07g065440*	11,6,7	6	AACAGTAATGCAGTTTCCTCAGG, CGACACAGTGAGAAGTCCGGAGG; CCTCCGCATGATTCACCTTCCGG, CCGCCTTTCGAATATTTCCAAGG; ACACTCTTAAGGAAAATCAAAGG, CCGCTGATCTACATCGAACGGTT	Micro-Tom
pYZ182	*TAM, REC8, SPO11-1*	*Solyc11g005090, Solyc06g074860-Solyc06g074870-Solyc06g074880, Solyc07g065430-Solyc07g065440*	11,6,7	3	CGACACAGTGAGAAGTCCGGAGG; CCTCCGCATGATTCACCTTCCGG; ACACTCTTAAGGAAAATCAAAGG	MbTMV-MT F1; Maxeza F1; Funtelle F1
Note: PAM sequences (CCN or NGG) were highlighted by red colours.						

pYZ1 has two guide RNAs per gene (2 gRNAs Vs *SPO11-1*, 2gRNAs Vs *REC8*, 2 gRNAs Vs *TAM*) and was used in the work in inbred Micro-Tom. pYZ182 has one guide RNA per gene (1 gRNA Vs *SPO11-1*, 1 gRNA Vs *REC8*, 1 gRNA Vs *TAM*) and was used in the work on MbTMV-MT F1, Maxeza F1 and Funtelle F1."

Supplementary Table 2. Alleles of *spo11-1*, *rec8* and *tam* in Micro-Tom background.

Mutant name	Mutation sites	sgRNA1 mutation	sgRNA2 mutation	CDS length (bp)	Protein length (aa)	Origin
spo11-1-1	+1 bp	ACACTCTTAAGGAAAATACAAAGG	CCGCTGATCTACATCGAACGGTT	78	25	CRISPR-Cas9 of *SlSPO11-1* (pYZ1)
spo11-1-2	-5 bp	ACACTCTTAAG-----TCAAAGG (-5 bp)	CCGCTGATCTACATCGAACGGTT	72	23	
WT *SPO11-1*	-	ACACTCTTAAGGAAAATCAAAGG	CCGCTGATCTACATCGAACGGTT	1152	383	Control
rec8-1	-180 bp (-103 bp of cDNA)	CCTCCG-A---------------	-----------------CCAAGG	1152	383	CRISPR-Cas9 of *SlREC8* (pYZ1)
rec8-2	-1 bp	CCTCCG-ATGATTCACCTTCCGg (-1 bp)	CCGCCTTTCGAATATTTCCAAGG	1200	399	
WT *REC8*	-	CCTCCGCATGATTCACCTTCCGG	CCGCCTTTCGAATATTTCCAAGG	1887	628	Control
tam-1	-1 bp	No editing	CGACACAGTGAGAAGT-CGGAGG	477	158	CRISPR-Cas9 of *SlTAM* (pYZ14)
tam-2	-208 bp	AACAGTAATGCAGTTTC------(-6 bp)	----------------------(-202 bp)GAGG	270	89	
tam-3	-63 bp	AACAGTAATGCA-----CTCAGG(-5 bp)	CGACACAGTGAGAAG--------(-58 bp)	324	107	
tam-4	-238 bp	AACAGTAATGCAG---------(-27 bp)	----------------------- (-211 bp)	384	127	
tam-5	+1 bp	No editing	CGACACAGTGAGAAGTCACGGAGG	483	160	
tam-6	-1 bp	No editing	CGACACAGTGAGAAGT-CGGAGG	477	158	CRISPR-Cas9 of *MiMe* population (pYZ1)
WT *TAM*	-	AACAGTAATGCAGTTTCCTCAGG	CGACACAGTGAGAAGTCCGGAGG	1473	490	Control

Note: PAM sequences (CCN or NGG) were highlighted by red colours. (*tam-6* has the same mutation with *tam-1*.)

Supplementary Table 3. Pollen size in Micro-Tom and five *tam* alleles by particle size detection.

Annotation	Wild type	*tam-1*	*tam-2*	*tam-3*	*tam-4*	*tam-5*
Number of sterile pollen (7.998-20.03 µm)	2289	2473	2588	2566	1907	2434
Number of reduced pollen (20.03-28.15 µm)	13051	5087	4494	5372	5547	5378
Number of unreduced pollen (28.15-40.23 µm)	331	5570	6473	3170	3820	4409
Percentage of unreduced pollen	0.02	0.42	0.48	0.29	0.34	0.36
Total number of pollen	15671	13130	13555	11108	11274	12221

**Supplementary Table 4. Mutation sites of different *MiMe* mutants.**					
Mutant name	*SPO11-1*	*REC8*	*TAM*	Background	Ploidy
MicroTom^{MiMe}	+1bp	-180bp	-1bp	MicroTom inbred line	Diploid
MbTMV-MT^{MiMe-A}	-1bp/-2bp	+1bp/-40bp	-1bp/-31bp	Moneyberg-TMV x Micro-Tom F1 hybrid line	Diploid
MbTMV-MT^{MiMe-B}	-1bp/-2bp	+1bp/-40bp	-1bp/-31bp	Moneyberg-TMV x Micro-Tom F1 hybrid line	Diploid
MbTMV-MT^{MiMe-C}	-1bp	-1bp/+1bp	-4bp/-5bp	Moneyberg-TMV x Micro-Tom F1 hybrid line	Diploid
MbTMV-MT^{MiMe-D}	-1bp/-2bp	+1bp/-40bp	-1bp/-31bp	Moneyberg-TMV x Micro-Tom F1 hybrid line	Diploid
Maxeza^{MiMe}	+1bp/-4bp	+1bp	+1bp/-1bp	Maxeza F1 hybrid line	Diploid
Maxeza^{MiMe-i}	+1bp/-6bp	+1bp	-1bp	Maxeza F1 hybrid line	Diploid
Maxeza$^{MiMe-ii}$	+1bp	+1bp	-4bp/-1bp	Maxeza F1 hybrid line	Diploid
Funtelle^{MiMe}	-1bp/-5bp	+1bp	-1bp/-2bp	Funtelle F1 hybrid line	Diploid
Funtelle^{MiMe-i}	-2bp/-7bp	+1bp	-1bp	Funtelle F1 hybrid line	Diploid
Funtelle$^{MiMe-ii}$	+1bp/-1bp	+1bp	-1bp/-27bp	Funtelle F1 hybrid line	Diploid
MbTMV-MT-Funtelle^{MiMe-1}	-1bp/-2bp/-1bp/-5bp	+1bp/-40bp	-1bp/-2bp/-31bp	Funtelle F1 & (Moneyberg-TMV x Micro-Tom F1)	Tetraploid
MbTMV-MT-Funtelle^{MiMe-2}	-1bp/-2bp/-1bp/-5bp	+1bp/-40bp	-1bp/-2bp/-31bp	Funtelle F1 & (Moneyberg-TMV x Micro-Tom F1)	Tetraploid
MbTMV-MT-Funtelle^{MiMe-3}	-1bp/-2bp/-1bp/-5bp	+1bp/-40bp	-1bp/-2bp/-31bp	Funtelle F1 & (Moneyberg-TMV x Micro-Tom F1)	Tetraploid
MbTMV-MT-Funtelle^{MiMe-4}	-1bp/-2bp/-1bp/-5bp	+1bp/-40bp	-1bp/-2bp/-31bp	Funtelle F1 & (Moneyberg-TMV x Micro-Tom F1)	Polyploid
MbTMV-MT-Funtelle^{MiMe-5}	-1bp/-2bp/-1bp/-5bp	+1bp/-40bp	-1bp/-2bp/-31bp	Funtelle F1 & (Moneyberg-TMV x Micro-Tom F1)	Tetraploid
MbTMV-MT-Maxeza^{MiMe-1}	+1bp/-2bp/-1bp/-4bp	+1bp/-40bp	-1bp/+1bp/-31bp	(Moneyberg-TMV x Micro-Tom F1) & Maxeza F1	Tetraploid
MbTMV-MT-Maxeza^{MiMe-2}	+1bp/-2bp/-1bp/-4bp	+1bp/-40bp	-1bp/+1bp/-31bp	(Moneyberg-TMV x Micro-Tom F1) & Maxeza F1	Tetraploid
MbTMV-MT-Maxeza^{MiMe-3}	+1bp/-2bp/-1bp/-4bp	+1bp/-40bp	-1bp/+1bp/-31bp	(Moneyberg-TMV x Micro-Tom F1) & Maxeza F1	Tetraploid
MbTMV-MT-Maxeza^{MiMe-4}	+1bp/-2bp/-1bp/-4bp	+1bp/-40bp	-1bp/+1bp/-31bp	(Moneyberg-TMV x Micro-Tom F1) & Maxeza F1	Tetraploid
MbTMV-MT-Maxeza^{MiMe-5}	+1bp/-2bp/-1bp/-4bp	+1bp/-40bp	-1bp/+1bp/-31bp	(Moneyberg-TMV x Micro-Tom F1) & Maxeza F1	Tetraploid
MbTMV-MT-Maxeza^{MiMe-6}	+1bp/-2bp/-1bp/-4bp	+1bp/-40bp	-1bp/+1bp/-31bp	(Moneyberg-TMV x Micro-Tom F1) & Maxeza F1	Tetraploid
MbTMV-MT-Maxeza^{MiMe-7}	+1bp/-2bp/-1bp/-4bp	+1bp/-40bp	-1bp/+1bp/-31bp	(Moneyberg-TMV x Micro-Tom F1) & Maxeza F1	Tetraploid
MbTMV-MT-Maxeza^{MiMe-8}	+1bp/-2bp/-1bp/-4bp	+1bp/-40bp	-1bp/+1bp/-31bp	(Moneyberg-TMV x Micro-Tom F1) & Maxeza F1	Polyploid
MbTMV-MT-Maxeza^{MiMe-9}	+1bp/-2bp/-1bp/-4bp	+1bp/-40bp	-1bp/+1bp/-31bp	(Moneyberg-TMV x Micro-Tom F1) & Maxeza F1	Tetraploid
MbTMV-MT-Maxeza$^{MiMe-10}$	+1bp/-2bp/-1bp/-4bp	+1bp/-40bp	-1bp/+1bp/-31bp	(Moneyberg-TMV x Micro-Tom F1) & Maxeza F1	Tetraploid
MbTMV-MT-Maxeza$^{MiMe-11}$	+1bp/-2bp/-1bp/-4bp	+1bp/-40bp	-1bp/+1bp/-31bp	(Moneyberg-TMV x Micro-Tom F1) & Maxeza F1	Tetraploid
MbTMV-MT-Maxeza$^{MiMe-12}$	+1bp/-2bp/-1bp/-4bp	+1bp/-40bp	-1bp/+1bp/-31bp	(Moneyberg-TMV x Micro-Tom F1) & Maxeza F1	Tetraploid
MbTMV-MT-Maxeza$^{MiMe-13}$	+1bp/-2bp/-1bp/-4bp	+1bp/-40bp	-1bp/+1bp/-31bp	(Moneyberg-TMV x Micro-Tom F1) & Maxeza F1	Tetraploid

**Supplementary Table 5. Raw hifiasm assembly statistics for Micro-Tom, Funtelle and Maxeza.**					
Genotype	Micro-Tom	Funtelle		Maxeza	
Haplotypes	N/A	Haplotype 1	Haplotype 2	Haplotype 1	Haplotype 2
Contigs	2739	1155	651	1488	689
Total length (Mb)	925.2	875.2	839.5	869.4	846.2
Max length (Mb)	52.6	76.12	69.815	73.183	74.75
N50 length (Mb)	21.1	56.5	41.3	55.7	39.4
N90 length (Mb)	0.077	1.3	1.8	2.1	4.6
L50	14	7	8	7	8
L90	229	29	34	25	27

‘N/A’ means ‘Not applicable’

Supplementary Table 6. Micro-Tom and MbTMV scaffolded chromosome statistics and quality metrics.		
**Accession**	**Micro-Tom**	**Moneyberg-TMV**
**Species**	S. lycopersicum	S. lycopersicum
**Reference**	This study	van Rengs et al., 2022
**Number of sequences**	12	12
**Number of sequences (>50kb)**	12	12
**Cumulative size (Mbp)**	812.44	824.45
**N50 (Mbp)**	67.8	68.5
**N90 (Mbp)**	56.8	54.7
**L50**	6	6
**L90**	11	11
**Longest sequence (Mbp)**	96.3	96.5
**Number of N's**	4000	0
**Number of internal N-regions**	40	0
**Raw LAI**	8.42	9.67
**LAI**	14.05	15.3
**Complete BUSCO (C)**	5851	5852
**Complete Single copy (S)**	5743	5747
**Complete Duplicated (D)**	108	105
**Fragmented (F)**	12	12
**Missing (M)**	87	86
**Total searched (solanales) busco v5.2.1**	5950	5950
**QV**	72.40	54.12
**Completeness**	99.22	99.10

Supplemental Table 7. Illumina sequencing reads per sample.

Sample	Library ID	Total Reads	Mapped Reads
MbTMV-MT_*MiMe-B*_offspring_3	S_5548_AA	191877456	191228378
MbTMV-MT_*MiMe-B*_offspring_4	S_5548_AB	177136359	176546007
MbTMV-MT_*MiMe-B*_offspring_5	S_5548_AC	153393423	152910134
MbTMV-MT_*MiMe-B*_offspring_6	S_5548_AD	172904626	172300274
MbTMV-MT_F1_1	S_5548_A	157132238	156522567
MbTMV-MT_F1_2	S_5548_B	155020768	154552488
MbTMV-MT_F1_3	S_5548_C	169617769	168974710
MbTMV-MT_F1_4	S_5548_D	164121342	163562427
MbTMV-MT_F1_5	S_5548_E	68727773	68459168
MbTMV-MT_F1_6	S_5548_F	143568799	143081665
MbTMV-MT_F2_1	S_5548_I	131734398	131365138
MbTMV-MT_F2_2	S_5548_J	141837932	141361403
MbTMV-MT_F2_3	S_5548_K	148372221	147973773
MbTMV-MT_F2_4	S_5548_L	99847316	99553819
MbTMV-MT_F2_5	S_5548_M	101835814	101458672
MbTMV-MT_F2_6	S_5548_N	108639330	108289257
MbTMV-MT_*MiMe-A*_offspring_1	S_5548_Q	184847673	184113982
MbTMV-MT_*MiMe-A*_offspring_2	S_5548_R	103877732	103551464
MbTMV-MT_*MiMe-A*_offspring_3	S_5548_S	160490176	159882359
MbTMV-MT_*MiMe-A*_offspring_4	S_5548_T	165688346	165086012
MbTMV-MT_*MiMe-A*_offspring_5	S_5548_U	163483681	162861442
MbTMV-MT_*MiMe-A*_offspring_6	S_5548_V	197858635	197078322
MbTMV-MT_*MiMe-B*_offspring_1	S_5548_Y	199730174	199082632
MbTMV-MT_*MiMe-B*_offspring_2	S_5548_Z	158852359	158408222
MbTMV-MT-Funtelle_1	S5693_A	138481538	138051807
MbTMV-MT-Funtelle_2	S5693_B	141422109	140984544
MbTMV-MT-Funtelle_3	S5693_C	139182747	138727697
MbTMV-MT-Funtelle_4	S5693_D	133859517	133199712
MbTMV-MT-Funtelle_5	S5693_E	125192153	124793213
MbTMV-MT-Maxeza_1	S5693_F	116389140	116151804
MbTMV-MT-Maxeza_2	S5693_G	150160273	149831723
MbTMV-MT-Maxeza_3	S5693_H	118497814	118247758
MbTMV-MT-Maxeza_4	S5693_I	140230644	139964309
MbTMV-MT-Maxeza_5	S5693_J	142523915	142253758
MbTMV-MT-Maxeza_6	S5693_K	137264255	136983857
MbTMV-MT-Maxeza_7	S5693_M	143339291	143026198
MbTMV-MT-Maxeza_8	S5693_N	122048965	121825911
MbTMV-MT-Maxeza_9	S5693_O	142865729	142563186
MbTMV-MT-Maxeza_10	S5693_P	138865705	138593286
MbTMV-MT-Maxeza_11	S5693_Q	136422525	136144027
MbTMV-MT-Maxeza_12	S5693_R	149042300	148756296
MbTMV-MT-Maxeza_13	S5693_S	153131061	152826944
Maxeza F1_1	S5693_T	134208096	134016219
Maxeza F1_2	S5693_U	123121727	122922069
Funtelle F1_1	S5693_V	133499118	133010225
Funtelle F1_2	S5693_W	142192383	141645542

Supplementary Table 8. Seed formation rate after hybridization of hybrid *MiMe* and control plants.

	Female	Male	Fruite weight (g)	Seed number
Control	Maxeza F1	MbTMV-MTV F1	79.78	68
			61.19	59
			68.38	49
			63.39	58
			54.11	57
	Funtelle F1	MbTMV-MTV F1	9.84	45
			7.79	38
			5.83	23
			6.81	31
			6.74	19
4-Hap	Maxeza^{MiMe}	MbTMV-MTV^{MiMe}	24.63	5
			17.52	3
			14.17	2
			29.96	6
			24.43	4
	Funtelle^{MiMe}	MbTMV-MTV^{MiMe}	6.02	3
			5.89	2
			4.51	1
			4.33	1
			4.97	1

Supplementary Table 9. List of oligonucleotides used in this study

Primer name	Oligo sequence (From 5' to 3' direction)	The usage of primers
M13F	TGTAAAACGACGGCCAGT	The identification of the *pDIRECT_22C* vector
TC320	CTAGAAGTAGTCAAGGCGGC	
AtCAS9-F	ATGCCACAGGTGAACATCGT	The identification of Cas9 in transgenic plants
AtCAS9-R	GAGAGCAAGCTCGTTTCCCT	
NPT-35S-F	TTCAGTGACAACGTCGAGCA	The identification of kanamycin resistance gene in transgenic plants
NPT-35S-R	GACGTAAGGGATGACGCACA	
CmYLCV	TGCTCTTCGCGCTGGCAGACATACTGTCCCAC	The primers used for the CRISPR-Cas9 vector construction
SPO11_2R_gRNA2	TGGTCTCCTCCTTAAGAGTGTCTGCCTATACGGCAGTGAAC	
SPO11_3F_gRNA2	TGGTCTCAAGGAAAATCAAGTTTTAGAGCTAGAAATAGC	
SPO11_1R_gRNA1	TGGTCTCCCATCGAACGGTTCTGCCTATACGGCAGTGAAC CTGCAGGAAG	
SPO11_2F_gRNA1	TGGTCTCAGATGTAGATCAGGTTTTAGAGCTAGAAATAGC	
REC8_3R_gRNA3	TGGTCTCCTCACCTTCCGGCTGCCTATACGGCAGTGAAC	
REC8_4F_gRNA3	TGGTCTCAGTGAATCATGCGGGTTTTAGAGCTAGAAATAGC	
REC8_4R_gRNA4	TGGTCTCCTTCGAAAGGCGGCTGCCTATACGGCAGTGAAC	
REC8_5F_gRNA4	TGGTCTCACGAATATTTCCAGTTTTAGAGCTAGAAATAGC	
TAM_5R_gRNA5	TGGTCTCCCTGCATTACTGTTCTGCCTATACGGCAGTGAAC	
TAM_6F_gRNA5	TGGTCTCAGCAGTTTCCTCGTTTTAGAGCTAGAAATAGC	
TAM_6R_gRNA6	TGGTCTCCTTCTCACTGTGTCGCTGCCTATACGGCAGTGAAC	
TAM_7F_gRNA6	TGGTCTCAAGAAGTCCGGGTTTTAGAGCTAGAAATAGC	
CSY_term	TGCTCTTCTGACCTGCCTATACGGCAGTGAAC	
SPO11-1-JC-3LP	GGTTGGTGGTGAGGTTCGAA	The identification of *SPO11-1* gene
SPO11-1-JC-4RP	AGCACTTCACTACGCATAACT	
REC8-JC-1LP	CTGTGCAGACTTTGAGTGT	The identification of *REC8* gene
REC8-JC-1RP	TGTTGGATGCAGTCCCATA	
TAM-JC-2LP	CTTGAGGTCATTTTTGGCT	The identification of *TAM* gene
TAM-JC-1RP	CTGCGGCTTTGACATGTTCT	
CU_SPO11_F1	TGTAAAACGACGGCCAGTTACAAACAACGTGAACCTGC	The identification of *SPO11-1* sgRNA1 by NGS
CU_SPO11_R1	CAGGAAACAGCTATGACGAACGCGAGAATCGTAATGC	
CU_SPO11_F4	TGTAAAACGACGGCCAGTACTCGTTCCATTGTTGAGGATC	The identification of *SPO11-1* sgRNA2 by NGS
CU_SPO11_R3	CAGGAAACAGCTATGACGCACTTCACTACGCATAACTTG	
CU_REC8_F2	TGTAAAACGACGGCCAGTTCACTATCCAGCACCACTCC	The identification of *REC8* sgRNA1 by NGS
CU_REC8_R1	CAGGAAACAGCTATGACAGGAGGAAAGGGTGGAGAG	
CU_REC8_F4	TGTAAAACGACGGCCAGTCCTTTCCTCCTGAACCATCC	The identification of *REC8* sgRNA2 by NGS
CU_REC8_R3	CAGGAAACAGCTATGACTGGTTCTGAGGTCCTCCATG	
CU_TAM_F1	TGTAAAACGACGGCCAGTGGCTTTGGAAGGCACCATG	The identification of *TAM* sgRNA1 by NGS
CU_TAM_R2	CAGGAAACAGCTATGACCTGCTTGGTCTCACAGAGG	
CU_TAM_F4	TGTAAAACGACGGCCAGTTTCCTGTTCCTGCCACTTG	The identification of *TAM* sgRNA2 by NGS
CU_TAM_R5	CAGGAAACAGCTATGACTACACGCTTTCTTCTCGATGG	
OSD1-JC-2LP	CAAATGGGTTTGCATAGAAG	The identification of *OSD1* gene
OSD1-JC-2RP	CCAGTTACTCTTGTAGTTCG	
OSD1_5R_gRNA5	TGGTCTCCCCGTCGACGACTGCTGCCTATACGGCAGTGAAC	The primers used for the CRISPR-Cas9 vector construction
OSD1_6F_gRNA5	TGGTCTCAACGGAGATCAAGTTTTAGAGCTAGAAATAGC	
OSD1_6R_gRNA6	TGGTCTCCGCTGTAGTCTGTACTGCCTATACGGCAGTGAAC	
OSD1_7F_gRNA6	TGGTCTCACAGCAGAAGCAGTTTTAGAGCTAGAAATAGC	
