# Supplementary material for: Harnessing clonal gametes in hybrid crops to engineer polyploid genomes
Source: Nat Genet. 2024 May 13;56(6):1075–9. doi: 10.1038/s41588-024-01750-6 (PMC11176054; doi:10.1038/s41588-024-01750-6)
Supplement: Supplementary file 2 — Reporting Summary [file 41588_2024_1750_MOESM2_ESM.pdf]

nature portfolio

Corresponding author(s): Charles Underwood

Last updated by author(s): Mar 22, 2024

Reporting Summary

Nature Portfolio wishes to improve the reproducibility of the work that we publish. This form provides structure for consistency and transparency in reporting. For further information on Nature Portfolio policies, see our Editorial Policies and the Editorial Policy Checklist.

Statistics

For all statistical analyses, confirm that the following items are present in the figure legend, table legend, main text, or Methods section.

n/a	Confirmed	
☐	☒	The exact sample size (*n*) for each experimental group/condition, given as a discrete number and unit of measurement
☐	☒	A statement on whether measurements were taken from distinct samples or whether the same sample was measured repeatedly
☐	☒	The statistical test(s) used AND whether they are one- or two-sided<br>*Only common tests should be described solely by name; describe more complex techniques in the Methods section.*
☐	☒	A description of all covariates tested
☐	☒	A description of any assumptions or corrections, such as tests of normality and adjustment for multiple comparisons
☐	☒	A full description of the statistical parameters including central tendency (e.g. means) or other basic estimates (e.g. regression coefficient) AND variation (e.g. standard deviation) or associated estimates of uncertainty (e.g. confidence intervals)
☐	☒	For null hypothesis testing, the test statistic (e.g. *F*, *t*, *r*) with confidence intervals, effect sizes, degrees of freedom and *P* value noted<br>*Give P values as exact values whenever suitable.*
☒	☐	For Bayesian analysis, information on the choice of priors and Markov chain Monte Carlo settings
☒	☐	For hierarchical and complex designs, identification of the appropriate level for tests and full reporting of outcomes
☒	☐	Estimates of effect sizes (e.g. Cohen's *d*, Pearson's *r*), indicating how they were calculated

Our web collection on statistics for biologists contains articles on many of the points above.

Software and code

Policy information about availability of computer code

Data collection	High throughput data of single pollen diameter was collected via a Multisizer 4e (Beckman Counter, Germany). Scanning electron microscopy (SEM) data was obtained by emission scanning electron microscope. Images of spread meiotic chromosomes were captured using a Zeiss Axio Imager Z2 upright microscope. Ploidy determination of plants was carried out using flow cytometry of leaf nuclei via DAPI staining. DNA sequencing was carried out on Illumina, MGI and PacBio HiFi platforms. Seeds from wild type and mutants were imaged using a Leica M205 FA digital stereomicroscope (Leica Microsystems, Germany). Leaf chlorophyll contents of control and 4-Hap plants were measured via a powerful tool AtLEAF (https://www.atleaf.com/, US).

Data analysis

Fruit, seed and pollen data analysis: Graphpad Prism 9, Leica Application Suite X v3.7.3.23245 (LAS X), ImageJ 1.51u (Fiji), Zeiss Labscope v3.1;
Phylogenetic tree: ClustalX2, MEGA11;
Chromosome data: ZEN 3.5 (blue edition);
Single pollen size: Multisizer 4e v4.04, Graphpad Prism 9;
Illumina sequencing data: CLC Main Workbench 21.0.5;
Ploidy determination and flow cytometry analysis: FCS Express 7, CytExpert v2.4.0.28 (Beckmann Counter, Germany)
Mutiple protein sequence analysis: ClustalX2, BioEdit v7.2.0;
Genome assemblies and downstream analysis: Hifiasm v0.16.1-r375, Salsa v2.2, Juicebox v1.11.08, Burrows-Wheeler Aligner v0.7, Samtools v1.9, Bedtools v2.30, minimap2 v2.24-r1122, D-GENIES v1.4.0, RagTag v2.1.0, genomescope v1.0;
Marker detection: bwa-mem v0.7.17 and minimap2 v2.24-r1122;
Determination of crossovers: GATK HaplotypeCaller v4.2.4.1;
Detection of genomics structural variations: SyRI v1.6.3.

For manuscripts utilizing custom algorithms or software that are central to the research but not yet described in published literature, software must be made available to editors and reviewers. We strongly encourage code deposition in a community repository (e.g. GitHub). See the Nature Portfolio guidelines for submitting code & software for further information.

Data

Policy information about availability of data

All manuscripts must include a data availability statement. This statement should provide the following information, where applicable:

- Accession codes, unique identifiers, or web links for publicly available datasets
- A description of any restrictions on data availability
- For clinical datasets or third party data, please ensure that the statement adheres to our policy

Raw sequencing data of MbTMV and the MbTMV genome assembly are available at the European Nucleotide Archive (ENA) under project numbers PRJEB44956 and PRJEB63089. Raw sequencing data of Micro-Tom (PRJEB62441), Funtelle (PRJEB62442) and Maxeza (PRJEB62443) are available at the ENA. Raw sequencing data of MbTMV-MT F1 hybrids, MbTMV-MT F2 offspring and all MiMe offspring (selfings and hybridizations) are available at the ENA under project number PRJEB63089.
Dryad Submission entitled "PacBio HiFi based haplotype-aware assemblies of tomato hybrid varieties Funtelle and Maxeza" with a unique digital object identifier (DOI): https://doi.org/10.5061/dryad.931zcrjs4
Dryad Submission entitled "A chromosome-scale de novo genome assembly of the dwarf tomato variety Micro-Tom" with a unique digital object identifier (DOI): https://doi.org/10.5061/dryad.h9w0vt4qd
The protein sequences were acquired from the Arabidopsis database TAIR (The Arabidopsis Information Resource, https://www.arabidopsis.org/) and then protein BLAST was performed against the phytozome database (https://phytozome-next.jgi.doe.gov/), the UniProt protein database (https://www.uniprot.org/blast), the Solanaceae Genomics Network database (https://solgenomics.net/) and the NCBI database (https://blast.ncbi.nlm.nih.gov/Blast.cgi) to identify homologous protein sequences in other species. Protein sequences alignment were achieved using Clustal X2 followed by construction of phylogenetic tree using MEGA11. Gene structure images were created using Exon-Intron Graphic Maker (http://wormweb.org/exonintron).

Research involving human participants, their data, or biological material

Policy information about studies with human participants or human data. See also policy information about sex, gender (identity/presentation), and sexual orientation and race, ethnicity and racism.

Reporting on sex and gender	NA
Reporting on race, ethnicity, or other socially relevant groupings	NA
Population characteristics	NA
Recruitment	NA
Ethics oversight	NA

Note that full information on the approval of the study protocol must also be provided in the manuscript.

Field-specific reporting

Please select the one below that is the best fit for your research. If you are not sure, read the appropriate sections before making your selection.

☒ Life sciences ☐ Behavioural & social sciences ☐ Ecological, evolutionary & environmental sciences

For a reference copy of the document with all sections, see nature.com/documents/nr-reporting-summary-flat.pdf

Life sciences study design

All studies must disclose on these points even when the disclosure is negative.

Sample size	We made use of one model hybrid (Moneyberg-TMV x Micro-Tom) and two commercial F1 hybrid lines (Funtelle and Maxeza) in this study without making a power calculation. By generating MiMe triple mutants in these three genetic backgrounds that represent highly divergent tomato genotypes we could demonstrate the MiMe phenotype was robust to different genetic backgrounds. The exact sample size for each experiment (single pollen particle size measurement, flow cytometry, embryo rescue, cytology) was clearly mentioned in the main text, figure legend or methods.
Data exclusions	No data were excluded.
Replication	Replications for each experiment were clearly stated in main text, figure legends or Methods section. For tam mutants, we tested five different alleles for experiments. At least three independent samples (biological replicates) and three replicate samples (technical replicates) were performed for all experiments.
Randomization	For the phenotype testing of F1 hybrid, F2 offspring and hybrid MiMe offspring, location of plants in the greenhouse was random. Meanwhile, all experiments plants were grown in consistent conditions, with appropriate controls grown side-by-side in Bronson Chamber, Percival Chamber and greenhouse.
Blinding	Where relevant blinding was carried out including during fruit weight analysis and other plant phenotyping experiments during data collection by technicians. The watering and plant nutrient irrigation system was performed without knowledge of plant genotype by greenhouse gardeners. In any non-blinded analysis several authors reviewed the data to ensure robust data analysis had been carried out.

Reporting for specific materials, systems and methods

We require information from authors about some types of materials, experimental systems and methods used in many studies. Here, indicate whether each material, system or method listed is relevant to your study. If you are not sure if a list item applies to your research, read the appropriate section before selecting a response.

Materials & experimental systems

n/a	Involved in the study	
☒	☐	Antibodies
☒	☐	Eukaryotic cell lines
☒	☐	Palaeontology and archaeology
☒	☐	Animals and other organisms
☒	☐	Clinical data
☒	☐	Dual use research of concern
☐	☒	Plants

Methods

n/a	Involved in the study	
☒	☐	ChIP-seq
☐	☒	Flow cytometry
☒	☐	MRI-based neuroimaging

Flow Cytometry

Plots

Confirm that:

☒ The axis labels state the marker and fluorochrome used (e.g. CD4-FITC).

☒ The axis scales are clearly visible. Include numbers along axes only for bottom left plot of group (a 'group' is an analysis of identical markers).

☐ All plots are contour plots with outliers or pseudocolor plots.

☐ A numerical value for number of cells or percentage (with statistics) is provided.

Methodology

Sample preparation	One piece of fresh young tomato leaf (2cm x 3mm) was chopped using a sharp razor blade in 550 µL Galbraith's buffer (45 mM MgCl2, 30 mM sodium citrate, 20 mM MOPS, 0.1% (v/v) Triton X-100, pH7.0) (Galbraith, D. W. et al. 1983). Next the slurry was passed through a 30-µm CellTrics green filter (REF: 04-0042-2316, Sysmex). Subsequently, 20 µL DAPI (100 µg/mL) was added to 500 µL filtered sample, followed by incubation for 15 minutes and run on the CytoFLEX V5-B5-R3 flow cytometer following manufacturer's instructions.
Instrument	CytoFLEX V5-B5-R3 flow cytometer
Software	CytExpert, FCS Express 7
Cell population abundance	The plant ploidy levels were determined from tomato leaf nuclei. In this ploidy checking experiment, cell sorting and

Cell population abundance

purification steps were not carried out. After stable peaks were formed, 10,000 events per sample were acquired in fast mode for each independent measurement.

Gating strategy

Gating was used to ensure that non-nuclear particles/debris with weak DAPI staining were not considered as plant nuclei. An example of the gating strategy used is presented in the Supplementary Figure 6.

☒ Tick this box to confirm that a figure exemplifying the gating strategy is provided in the Supplementary Information.
